# Genetic Influence on Frequencies of Myeloid-Derived Cell Subpopulations in Mouse

**DOI:** 10.3389/fimmu.2021.760881

**Published:** 2022-01-26

**Authors:** Imtissal Krayem, Yahya Sohrabi, Eliška Javorková, Valeriya Volkova, Hynek Strnad, Helena Havelková, Jarmila Vojtíšková, Aigerim Aidarova, Vladimír Holáň, Peter Demant, Marie Lipoldová

**Affiliations:** ^1^ Laboratory of Molecular and Cellular Immunology, Institute of Molecular Genetics of the Czech Academy of Sciences, Prague, Czechia; ^2^ Department of Cell Biology, Faculty of Science, Charles University, Prague, Czechia; ^3^ Department of Nanotoxicology and Molecular Epidemiology, Institute of Experimental Medicine of the Czech Academy of Sciences, Prague, Czechia; ^4^ Department of Genomics and Bioinformatics, Institute of Molecular Genetics of the Czech Academy of Sciences, Prague, Czechia; ^5^ Department of Molecular and Cellular Biology, Roswell Park Comprehensive Cancer Center, Buffalo, NY, United States

**Keywords:** myeloid-derived cells, genetic control, CD11b^+^Gr1^+^ subpopulation, neutrophils, relative spleen weight, spleen architecture, candidate gene

## Abstract

Differences in frequencies of blood cell subpopulations were reported to influence the course of infections, atopic and autoimmune diseases, and cancer. We have discovered a unique mouse strain B10.O20 containing extremely high frequency of myeloid-derived cells (MDC) in spleen. B10.O20 carries 3.6% of genes of the strain O20 on the C57BL/10 genetic background. It contains much higher frequency of CD11b^+^Gr1^+^ cells in spleen than both its parents. B10.O20 carries O20-derived segments on chromosomes 1, 15, 17, and 18. Their linkage with frequencies of blood cell subpopulations in spleen was tested in F_2_ hybrids between B10.O20 and C57BL/10. We found 3 novel loci controlling MDC frequencies: *Mydc1, 2*, and *3* on chromosomes 1, 15, and 17, respectively, and a locus controlling relative spleen weight (*Rsw1*) that co-localizes with *Mydc3* and also influences proportion of white and red pulp in spleen. *Mydc1* controls numbers of CD11b^+^Gr1^+^ cells. Interaction of *Mydc2* and *Mydc3* regulates frequency of CD11b^+^Gr1^+^ cells and neutrophils (Gr1^+^Siglec-F^-^ cells from CD11b^+^ cells). Interestingly, *Mydc3*/*Rsw1* is orthologous with human segment 6q21 that was shown previously to determine counts of white blood cells. Bioinformatics analysis of genomic sequence of the chromosomal segments bearing these loci revealed polymorphisms between O20 and C57BL/10 that change RNA stability and genes’ functions, and we examined expression of relevant genes. This identified potential candidate genes *Smap1, Vps52, Tnxb*, and *Rab44.* Definition of genetic control of MDC can help to personalize therapy of diseases influenced by these cells.

## Introduction

Disruption of the normal hematological phenotypes is directly related to multiple diseases (1). Hematological traits have been associated with an increased risk for a number of clinical disorders such as cancer, autoimmune diseases, and total mortality (2). White blood cell (WBC) numbers are partly under genetic control, with heritability approximately 40%–60% (3, 4). Peripheral WBC levels vary among ethnic groups, with neutrophil number levels higher in European Americans than in African Americans (2, 5) due to mutation in Duffy antigen/chemokine receptor (*DARC*) gene (6). Different strains of mice exhibited different numbers of WBC (7–9), indicating that the resting state WBC counts are under genetic control. Hence, it is essential to identify genes controlling the elements of homeostasis of normal human and animal immune systems, including the relative frequencies of WBC subsets (10).

Genome-wide association studies (GWAS) identified the quantitative trait loci (QTL) controlling the homeostasis of WBC classes in human (11, 12) and mice (8, 13) (**Table 1**). However, identifying the genes underlying these variations remains challenging, as most detected QTLs are either in non-coding regions or in linkage disequilibrium with many other variants (24).

**Table 1:** QTLs controlling circulating WBC levels in human, mice and swine.

Trait	QTL, marker or position	Species	Summary
**Eosinophils proportion in circulating blood**	2q33 (D2S117–D2S434)	Human	The study was performed in 12-, 14-, and 16-year-old Australian twins in order to identify candidate genes involved with asthma pathophysiology (14).
**Eosinophil proportion in circulating blood**	5q31-33 (D5S500–D5S658)	Human	The study was performed in families where both parents are non-Hispanic white (15).
**Total WBC count**	1q23 (*DARC*),	Human	The study performed meta-analysis of data of 16,388 African-American participants in 7 cohort studies. Some of these results were replicated in three other ethnic groups (Hispanic Americans, Japanese and European Americans) (16)
	4q13 (*CXCL2*),		
	7q21 (*CDK6*),		
	17q21 (*PSMD3-CSF3*);		
**Neutrophil and monocyte count**	1q23 (*DARC*)		
**WBC count**	6p21.33, 17q21.1;	Human	The study performed meta-analysis of data of 19,509 participants in 7 cohort studies and 11,823 participants in 10 replication cohorts (17).
**Neutrophil count**	17q21.1;		
**Basophil count**	3q21.3;		
**Lymphocyte count**	6p21.33, 19p13.11;		
**Monocyte count**	2q31.3, 3q21.3, 8q24.21, 9q31.3;		
**Neutrophil count**	Chr7,92246306,	Human	The study performed a large-scale GWAS of 14,792 Japanese participants in the BioBank Japan Project. Some of these results were replicated in the cohorts of Caucasian populations (18).
	Chr17,35410238;		
**Monocyte count**	Chr2,182031910,		
	Chr6,31329647,		
	Chr8,130641292,		
	Chr14,24573639;		
**Basophil count**	Chr1,203942886,		
	Chr3,129799125,		
	Chr11,89515085,		
	Chr21,38774421;		
**Eosinophil count**	Chr3,129799125,		
	Chr6,31589278,		
	Chr6,135464902		
**WBC count**	Chr1,159062436,	Human	The study performed meta-analysis of data from Japanese, African-American, and European-American cohorts. The study replicated 10 previously known loci [including the loci identified by Reiner et al., 2011 (16)] and identified six new loci (19).
	Chr2,219099484,		
	Chr2,113841030,		
	Chr4,74977837,		
	Chr6,31247203,		
	Chr6,135426573,		
	Chr7,92408370,		
	Chr8,130597585,		
	Chr17,38156712;		
**Monocyte count**	Chr2,182319301,		
	Chr2,182323665,		
	Chr3,128297569,		
	Chr5,76058509,		
	Chr6,31221668,		
	Chr8,130624105,		
	Chr9,113915905;		
**Neutrophil count**	Chr4,74967890,		
	Chr6,32217092,		
	Chr7, 92408370,		
	Chr17, 38156712,		
	Chr17,38166879		
**Monocyte count**	9q31	Human	The study was performed on Australian Dutch individuals as part of a GWAS study to identify QTL for hematology traits (20).
**Counts of Baseline WBC**	*Wbcq1* (D1Mit282),	Mouse	Analysis was performed in whole blood from intercrosses between mouse strains NZW/LacJ, SM/J, and C57BLKS/J (9).
	*Wbcq2* (D3Mit142),		
	*Wbcq3* (D15Mit13),		
	*Wbcq4* (D1Mit306),		
	*Wbcq5* (D1Mit227),		
	*Wbcq6* (D14Mit98)		
**WBC**	Chr1,26971726,	Mouse	Analysis was performed on 100 inbred strains of the Hybrid mouse diversity panel by GWAS (8).
	Chr6,135927582,		
	Chr8,8119195,		
	Chr11,63825134,		
	Chr12,79259640,		
	Chr15,99555171,		
	Chr16,15916062,		
	Chr18,70410404;		
**WBC counts**	*SSC6* (DIAS0004496);	Swine	The study was performed on 843 Italian large white pigs by three GWAS scan approaches (single-trait, multi-trait, and Bayesian) analyzing 30 blood parameters (21, 22).
**Lymphocyte counts**	*SSC2* (DIAS0001270);		
**Neutrophil counts**	*SSC4* (MARC0052177);		
**Eosinophil counts**	*SSC3* (H3GA0009277,		
	H3GA0010692),		
	*SSC7* (H3GA0021970,		
	INRA0028736),		
	*SSC10* (H3GA0030197);		
**Basophils counts**	*SSC14* (ALGA0079529,		
	MARC0090899);		
**Monocyte counts**	*SSC15* (ALGA0084320)		
**Baseline levels of WBC**	*SSC7*(60cM), *SSC12*(32cM),	Swine	The study analyzed the QTL associated with leucocytes and platelet related traits in F_2_ of White Duroc X Erhualian pigs (23).
	*SSC15*(87cM);		
**Lymphocytes**	*SSC7*(59cM),		
	*SSC12*(26cM),		
	*SSC15*(97cM),		
**Neutrophils**	*SSC7*(62cM);		

CDK6: cyclin-dependent kinase 6; Chr.: chromosome; CSF3: colony stimulating factor 3 (granulocyte); CXCL2: chemokine (C-X-C motif) ligand 2; PSMD3: proteasome (prosome, macropain) 26S subunit, non-ATPase, 3; SSC: Sus scrofa (ssc; swine); Wbcq: white blood cells QTL

We examined the WBC subpopulations in spleens of the strain C57BL/10-*H2^pz^* (B10.O20), a *H2* semi-congenic strain on the C57BL/10 (B10) background carrying the O20/A (O20)-derived *H2^pz^* haplotype (25). The strain B10.O20 inherited from O20 also an additional 3.6% of its genome. Surprisingly, the myeloid-derived cell (MDC) frequencies in spleens of B10.O20 exceeded those of its two parental strains. To map the genes controlling these differences, we analyzed F_2_ hybrids between strains B10.O20 and B10, identified three loci on chromosomes 1, 15, and 17 and a suggestive linkage on chromosome 18 controlling MDC frequencies and relative spleen weight, and described potential candidate genes.

## Materials and Methods

### Mice

Female mice of strains O20 (*n* = 10), B10.O20 (*n* = 11), and B10 (*n* = 9) and F_2_ hybrids between B10.O20 and B10 (B10xB10.O20 [*n* = 78] and B10.O20xB10 [*n* = 190]) in two independent experiments were tested. Unequal numbers of mice in different crosses were due to difficulties in breeding of the cross B10xB10.O20. Mice were produced and housed in SPF conditions at the animal facility of the Institute of Molecular Genetics of the Czech Academy of Sciences and were, on average, 12 weeks old (median 12 weeks, min 8 weeks, max 18 weeks). Mice were killed by cervical dislocation and spleens were divided into four equal quarters for further analysis. The first part was used for immunophenotyping, the second for morphological analysis, and the third for expression analysis. The fourth part was kept as a reserve. All experiments were approved by the Ethical Committee of the Institute of Molecular Genetics of the Czech Academy of Sciences.

### Relative Spleen Weight

Spleen and total body weights were determined using the balance Adventurer-Pro (OHAUS Corporation, Pine Brook, NJ USA; Made in Switzerland), resolution *d* = 0.01 g. The relative spleen weight was calculated as spleen-to-body weight ratio × 1000.

### Immunophenotyping

One spleen quarter was homogenized in phosphate-buffered saline (PBS) using disposable pestles. Single-cell suspensions were washed in PBS containing 0.5% bovine serum albumin and incubated for 30 min on ice with the anti-mouse mAb against CD11b, CD14, F4/80, CD40, Gr1, CD3, CD4, CD8, and CD19; for details, see **Supplementary Table 1**. All samples were incubated with Pacific Blue-labeled anti-TER-119 to exclude erythroid cells. Dead cells were stained with Hoechst 33258 (Invitrogen). Fifty thousand events were acquired on a LSRII cytometer (BD Biosciences) and analyzed using FlowJo 9.9.3 (BD Biosciences).

### Genotyping of F_2_ Mice

DNA was isolated from tails using standard proteinase K procedure. B10.O20 strain differs from B10 at O20-derived regions on four chromosomes. These differential regions were typed using 5 microsatellite markers (D17Mit197, D17Mit21, D17Mit10, D17Mit66, and D18Mit24) and 2 SNP sites: chromosome 1, rs23555388 and chromosome 15, rs78065633 (Generi Biotech, Czech Republic). DNA was amplified by PCR as previously described (26). We detected the presence of allele-specific SNP sites on chromosomes 1 and 15 by digesting the amplicons with the restriction enzymes *Mwo*I (New England BioLabs, Inc.) and *Alu*I (Thermo Fisher Scientific, Inc.), respectively.

### RNA Isolation and RT-PCR Analysis

RNA was prepared by lysing a quarter of spleen stored at −80°C in TRI reagent (Sigma Aldrich). One microgram of RNA was treated with DNase (Promega, M6101) and then reverse transcribed and amplified as previously described (27) in a total volume of 10 μl. In detail, 1 µg of RNA was treated with DNase (Promega, M6101) and then reverse transcribed using 100 units of M-MLV Reverse Transcriptase (Sigma, M1302) with 1xMLV reverse transcriptase buffer, 1.4 µM of random hexamers (Thermo Fisher, N8080127), 2.5 units of ribonuclease inhibitor (Thermo Fisher, 15518012), and 5 mM of each dNTP (Sigma, DNTP100) per sample to obtain cDNA. cDNA was then diluted five times and 3 µl was used for amplification by 45 cycles of PCR (3 min denaturation at 95°C, 15 s denaturation at 95°C and 60 s annealing/extension at 60°C with a single fluorescence acquisition point repeated 45 times, and a melt curve program of 55°C to 95°C with 0.5°C increment with continuous fluorescence acquisition) using primers for the genes of interest and iQ SYBR Green Supermix (Bio-RAD, 1708882) for quantification. Primers (**Supplementary Table 2**) were designed by Quantprime (28) and purchased from Generi Biotech, Czech Republic. GAPDH is used as an internal control. Reactions were performed in a 384-well plate in LC480II light cycler (Roche Molecular Systems, Inc.) The average Ct values (cycle threshold) were used for quantification, and the relative expression was calculated [ratio (reference/target) = 2^(Ct(reference) − Ct(target))].

### Morphological Analysis of the Spleen

Another spleen quarter was processed for histology overnight using an automated vacuum tissue processor (Leica ASP200S) and embedded in paraffin using Leica EG1150H. Three-micrometer serial sections were prepared (Leica RM2255), stained with hematoxylin and eosin, and observed under light microscope Leica DM6000 at 10× magnification using the software LAS X, 64 bit. The brightness and contrast of the pictures were then adjusted using FIJI (29). The area of the white pulps was measured using the ellipse formula a*b*π where “a” is the major radius and “b” is the minor radius of the white pulp. The recorded area of one sample represents the average area of ten white pulps measured three times each. Selected samples were from mice 14 weeks old in average (median 14 weeks, min 13 weeks, max 16 weeks).

### Detection of Polymorphisms That Change RNA Stability and Genes’ Functions

We have sequenced the genomes of strains C57BL/10 and O20 using next-generation sequencing (NGS) system HiSeq 2500 (Illumina) (12× coverage). Processing, alignment, sorting and indexing of NGS data, variants filtration, annotation, and effect prediction were performed as described elsewhere (30). In detail, NGS data were preprocessed using software Trimmomatic (31) and overlapping pair reads were joined by software Flash (32). Alignment-reference mouse sequence mm10 (build GRCm38) was performed using BWA (Burrows-Wheeler Aligner) program (33). Mapped reads were sorted and indexed, and duplicated reads were marked. Local realignment around indels, base recalibration, and variants filtration were performed using software GATK (the Genome Analysis Toolkit) (34). IGV (Integrated Genome Viewer) (35) was used for visualization of results. Variant annotation and effect prediction was performed by software SnpEff (36). Protein variation effect predictions were performed by software PROVEAN (Protein Variation Effect Analyzer) (37). Analysis of conservation scores was performed using ConSurf software (38–40).

### Statistical Analysis

Differences between parental strains and between mice within parental strains were analyzed by Mann–Whitney test using the program Statistica for Windows 12.0 (StatSoft, Inc., Tulsa, Oklahoma, USA).

In F_2_ hybrids, variance components and mixed-model ANOVA of Statistica with genotype (marker) and grandparent-of-origin effect as fixed factors and age as a covariate were used to evaluate the role of genetic factors controlling the frequency of cell subpopulations and the relative spleen weight. When necessary for analysis by ANOVA, the original values of an analyzed parameter were transformed for normalization of the distribution as described in the legends to the tables. Markers and interactions with *p* < 0.05 were combined in a single comparison. All obtained nominal *p*-values were corrected for multiple testing by Bonferroni correction.

ANOVA or *t*-tests (as indicated) were used in GraphPad (version 5.04) to evaluate the effect of genetic factors controlling the expression level of potential candidate genes and size of the white pulp.

## Results

### Combination of Genomes of Two Parental Strains Gives Rise to a Strain Exceeding Hematological Parameters of Both of Them

**Figure 1:**
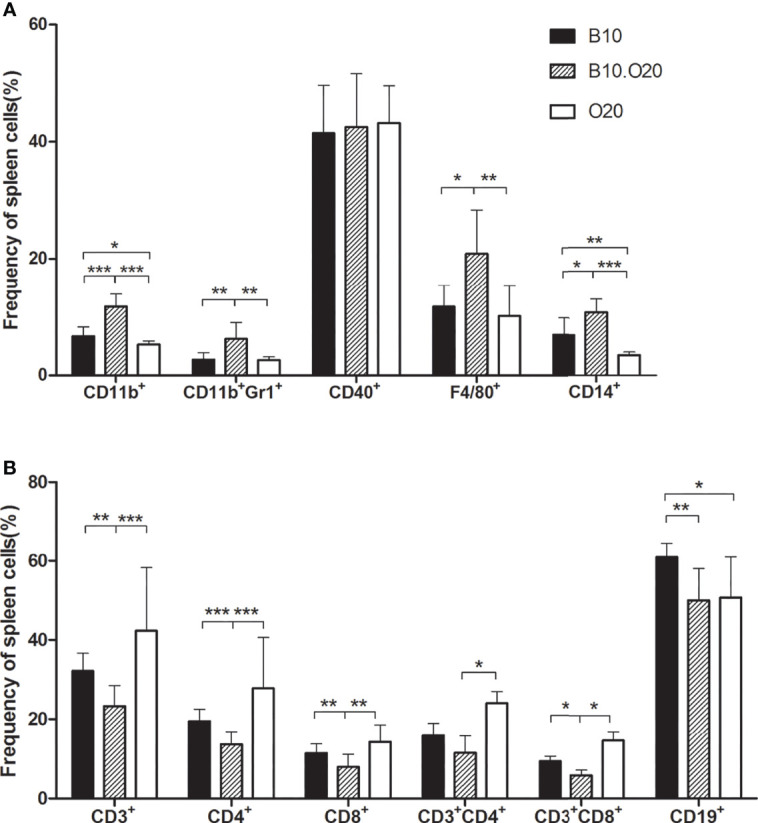
White blood cell subsets in spleens of strain B10.O20 and the parental strains C57BL/10 (B10) and O20. **(A)** The proportion of CD11b^+^, CD11b^+^Gr1^+^, CD40^+^, F4/80^+^, and CD14^+^ myeloid cell subsets. **(B)** The proportion of CD3^+^, CD4^+^, CD8^+^, CD3^+^CD4^+^, and CD3^+^CD8^+^ T-cell lineages, and CD19^+^ B-cell lineages. Values represent the average levels of samples tested in triplicates +SEM. Strains B10 (n = 9), B10.O20 (*n* = 11), and O20 (*n* = 10) are represented by black, striped, or white bars respectively. *p < 0.05; **p < 0.01; ***p < 0.001 (analyzed by Mann–Whitney test).

We analyzed frequencies of the main myeloid and lymphoid subpopulations in spleens of mice of the parental strains B10 and O20, and of recombinant strain B10.O20. For characterization of myeloid cell population, we examined markers F4/80, CD11b, and CD14 to characterize macrophage lineage, co-expression of CD11b and Gr1 to characterize granulocytes, and CD40 as a marker leukocyte with antigen-presenting function. For the characterization of T-cell lineage, we used a marker CD3 and markers CD4 and CD8 to distinguish the main CD3 subpopulations. Since CD4 molecule can be also expressed by other small cell populations, we also examined the presence of CD3^+^CD4^+^ (helper T cells) and CD3^+^CD8^+^ (cytotoxic T cells) subpopulations. To characterize cells of the B-cell lineage, we used B-cell marker CD19, which distinguishes B-cell lineage from T cells (41). Representative dot plots of myeloid and lymphoid cell subpopulations are shown in **Supplementary Figures 1, 2**, respectively. Frequencies of CD11b^+^, CD11b^+^Gr1^+^, CD14^+^, and F4/80^+^ cells in B10.O20 mice were about double their frequency in the parental strains B10 and O20, while the frequencies of CD3^+^, CD4^+^, CD8^+^, and CD3^+^CD8^+^ were significantly lower. Also, frequency of CD11b^+^, CD14^+^, and CD19^+^ cells differed between the strains B10 and O20. Levels of CD3^+^CD4^+^ cells in the strain B10.O20 differed significantly from the strain O20. The frequency of CD19^+^ cells was similar in both B10.O20 and O20, but lower than in the strain B10. Levels of CD40^+^ cells were not significantly different in the three strains (**Figure 1**).

### Loci Controlling Differences in MDC Frequencies

**Figure 2:**
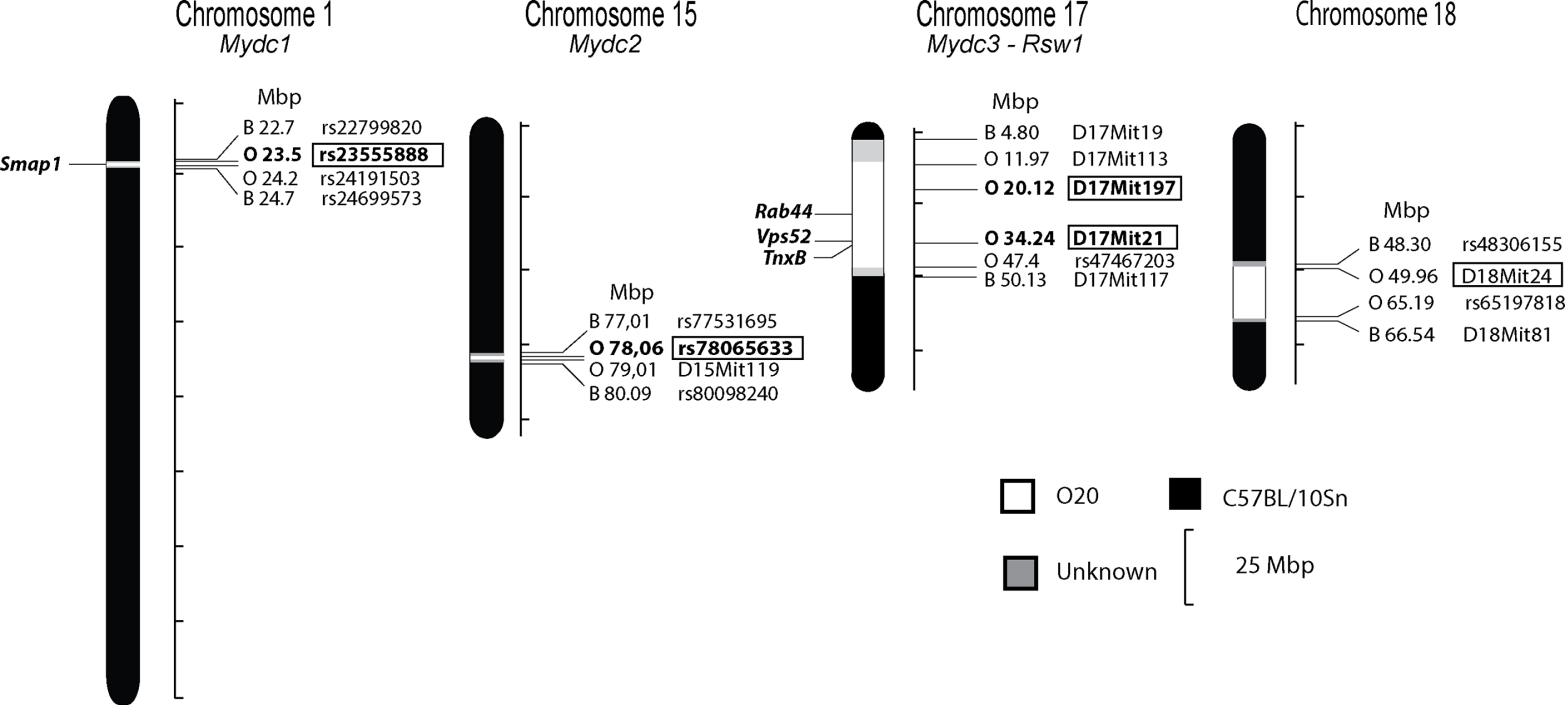
Positions of the loci that control relative spleen weight and frequencies of myeloid-derived subpopulations in spleen of the strain B10.O20. The regions of C57BL/10 and O20 are represented as black and white, respectively; the boundary regions of undetermined origins are shaded. The identified loci *Mydc1-3* and *Rsw1* and potential candidate genes are indicated. Only the markers or SNPs defining the boundaries of O20-derived segments and markers that were tested for linkage are shown (except syntenic D17Mit10). Bold in box—significant linkage, regular font in box—suggestive linkage.

Subsequently we used F_2_ hybrids between B10 and B10.O20 in order to map the genes controlling the frequencies of immune cell subsets in the strain B10.O20 and the relative spleen weight. We measured the frequencies of CD11b^+^, CD11b^+^Gr1^+^, CD11b^+^Ly6C^+^, CD11b^+^Ly6G^+^, CD11b^+^Siglec-F^+^, CD19^+^, and CD40^+^ cells, and the levels of Gr1^+^Siglec-F^-^ cells from CD11b^+^ cells (hereafter noted as neutrophils) and eosinophils (Gr1^-^Siglec-F^+^ cells from CD11b^+^ cells) by flow cytometry. We genotyped the O20-derived segments in F_2_ mice to detect the loci linked with cell subpopulation frequencies and analyzed the results by one-way ANOVA. **Figure 2** and **Table 2** summarize the loci controlling several phenotypes observed in the strain B10.O20.

**Table 2:** Summary of loci controlling spleen cell subsets in B10.O20.

Phenotype	Locus	Chr.	Marker	Cross	*p*	Bonf. corr. *p*
CD11b^+^Gr1^+^	*Mydc1*	1	rs23555388	B10xB10.O20	0.005	0.039
Relative spleen weight	*Rsw1*	17	D17Mit197 D17Mit21	Both crosses Both crosses	0.00002 0.00006	0.0001 0.0004
CD11b^+^Gr1^+^	Mydc2 *Mydc3	15*17	rs78065633 *D17Mit197	B10xB10.O20	0.001	0.008
Neutrophils (Gr1^+^Siglec-F^-^ cells from CD11b^+^ cells)	Mydc2 *Mydc3	15*17	rs78065633 *D17Mit197	B10xB10.O20	0.006	0.048
Neutrophils^#^	#	18	D18Mit24	B10.O20xB10	0.009	0.063

*interaction between loci; ^#^suggestive linkage.

**Figure 3:**
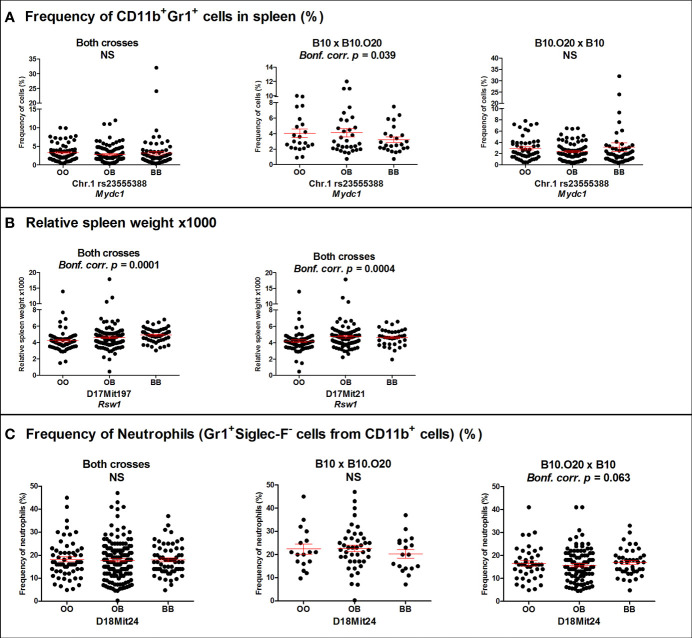
Genetic influence on frequency of **(A)** CD11b^+^Gr1^+^ cells, **(B)** relative spleen weight, and **(C)** neutrophils. Individual F_2_ hybrid mice between strain B10.O20 and B10 are shown. Means ± standard error mean (red lines) and *p*-values were calculated by analysis of variance (ANOVA). O and B indicate the presence of O20 and B10 allele, respectively. NS, Not significant.

**Figure 4:**
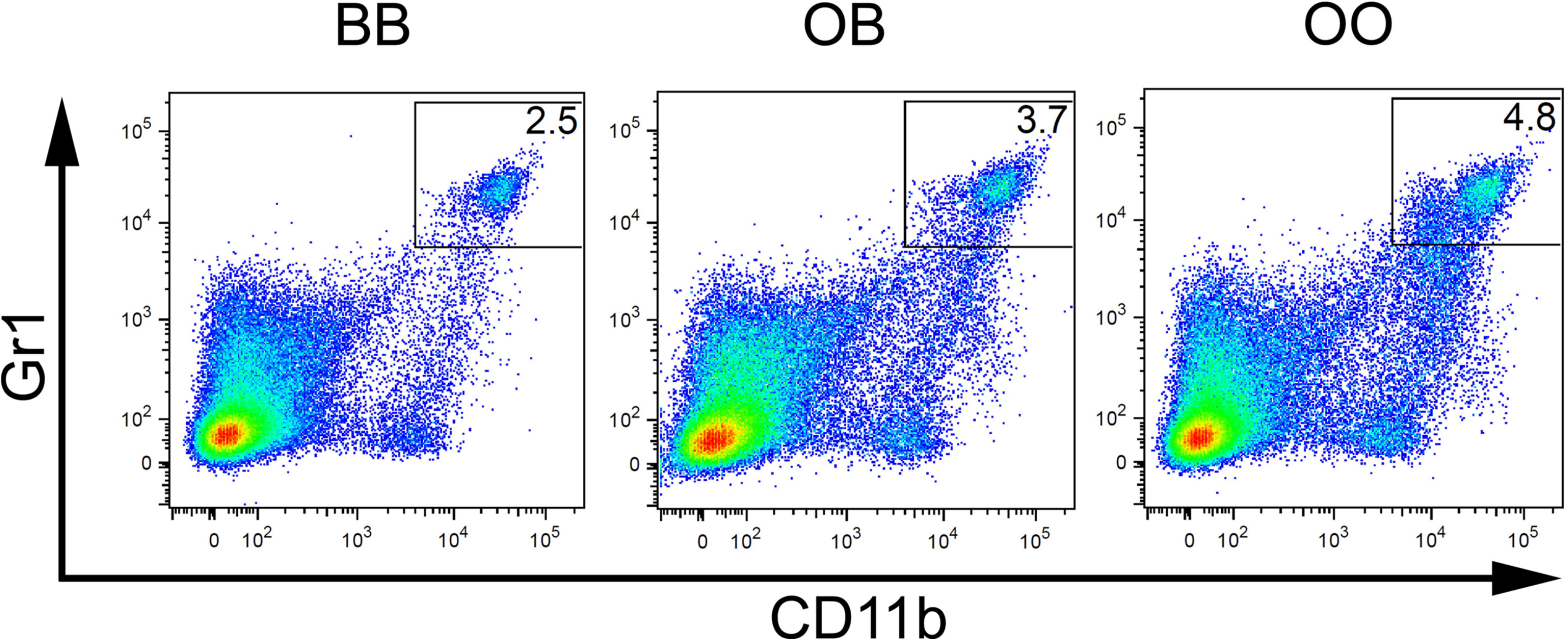
Genetic influence on frequency of CD11b^+^Gr1^+^ cells. Flow cytometry analysis of spleens of representative mice with BB, OB, and OO genotypes showing the Gr1/CD11b cell surface marker status of individual cells. O and B indicate the presence of O20 and B10 allele, respectively.

Loci *Mydc1* (Myeloid-derived cells 1) on chromosome 1, *Rsw1* (Relative spleen weight 1) on chromosome 17, and the suggestive locus on chromosome 18 exhibit a single gene effect (**Table 3** and **Figures 3**, **4**). Locus *Mydc1* linked with rs23555388 influences the frequency of the CD11b^+^Gr1^+^ subpopulation (Bonferroni corr. *p* = 0.039). Homozygotes in O20 allele (OO) exhibit higher numbers of CD11b^+^Gr1^+^ cells in spleen. The effect of this locus was observed only in the cross between B10 females and B10.O20 males, but no significant interaction between cross and SNP marker was observed. Although linkage for the cross B10.O20xB10 was not significant, phenotypes were concordant with the B10xB10.O20 cross, with OO genotype being the highest and BB genotype being the lowest. Locus *Rsw1* is linked to the markers D17Mit197 (Bonferroni corr. *p* = 0.0001) and D17Mit21 (Bonferroni corr. *p* = 0.0004). Mice homozygous in B10 (BB) allele of this locus show higher relative spleen weight. We have detected a suggestive linkage of neutrophil subpopulation with marker D18Mit24 (Bonferroni corr. *p* = 0.063). Heterozygotes in this locus had lower frequency of this subpopulation. The effect of this locus was observed only in the cross between B10.O20 females and B10 males, but no significant interaction between cross and genetic marker was observed.

**Table 3:** Loci controlling frequencies of myeloid-derived spleen cells and relative spleen weight in F_2_ hybrids between B10.O20 and B10.

Phenotype	Locus	Cross	Marker	Genotype									*p*	Bonf. corr. *p*
				BB			OB			OO				
CD11b^+^Gr1^+^	***Mydc1***	Both	rs23555388 (chr.1)	**2.16**	1.17	± 0.02	**2.72**	1.22	± 0.02	**2.91**	1.24	± 0.02	NS	NS
						(*n* = 72)			(*n* = 111)			(*n* = 70)		
		B10xB10.O20	rs23555388 (chr.1)	**2.42**	1.19	± 0.04	**4.16**	1.33	± 0.03	**4.18**	1.33	± 0.03	0.005	**0.039**
						(*n* = 23)			(*n* = 31)			(*n* = 22)		
		B10.O20xB10	rs23555388 (chr.1)	**1.75**	1.12	± 0.03	**1.93**	1.14	± 0.02	**2.41**	1.19	± 0.03	NS	NS
						(*n* = 49)			(*n* = 80)			(*n* = 48)		
Relative spleen weight	***Rsw1***	Both	D17Mit197	**4.85**	1.27	± 0.01	**4.45**	1.25	± 0.00	**4.01**	1.23	± 0.00	0.00002	**0.0001**
						(*n* = 53)			(*n* = 130)			(*n* = 73)		
		Both	D17Mit21	**4.71**	1.26	± 0.01	**4.50**	1.25	± 0.00	**4.00**	1.23	± 0.00	0.00006	**0.0004**
						(*n* = 64)			(*n* = 117)			(*n* = 75)		
Neutrophils (Gr1^+^Siglec-F^-^ cells from CD11b^+^ cells)	NN	Both	D18Mit24	**18.66**	4.32	± 0.12	**17.11**	4.14	± 0.08	**19.74**	4.44	± 0.12	NS	NS
						(*n* = 60)			(*n* = 137)			(*n* = 58)		
		B10xB10.O20	D18Mit24	**19.51**	4.42	± 0.21	**22.36**	4.73	± 0.14	**21.58**	4.65	± 0.22	NS	NS
						(*n* = 18)			(*n* = 41)			(*n* = 17)		
		B10.O20xB10	D18Mit24	**16.79**	4.10	± 0.13	**13.90**	3.73	± 0.09	**17.22**	4.15	± 0.14	0.009	0.063
						(*n* = 42)			(*n* = 96)			(*n* = 41)		

Means, standard error of mean (SEM) and p-values were calculated by analysis of variance (ANOVA). In order to obtain normal distribution required for ANOVA, the following transformations were used: CD11b^+^Gr1^+^ (% in spleen homogenates) - power of 5; % of Gr1^+^Siglec-F^-^ cells from CD11b^+^ cells – power of 2; relative spleen weight ([spleen weight/body weight] × 1000] - power 1/0.15. Transformed means ± SEM are shown next to average non-transformed mean values in bold. Only p-values significant or suggestive after Bonferroni correction are given. O and B indicate the presence of O20 and B10 allele, respectively. NS—Not significant. NN—not named.

**Figure 5:**
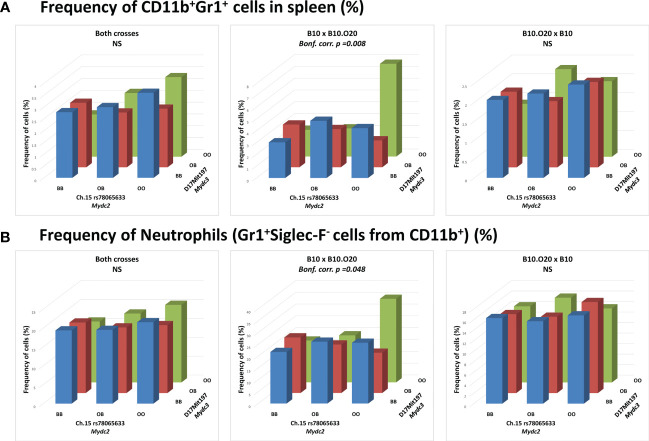
Interaction between loci *Mydc2* and *Mydc3* in control of myeloid-derived cells. Bars indicate the average frequency of **(A)** CD11b^+^Gr1^+^ cells or **(B)** neutrophils (Gr1^+^Siglec-F^-^ cells from CD11b^+^ cells) for the indicated cross and genotype. *p-*values were calculated by analysis of variance (ANOVA). O and B indicate the presence of O20 and B10 allele, respectively. NS, Not significant.

**Figure 6:**
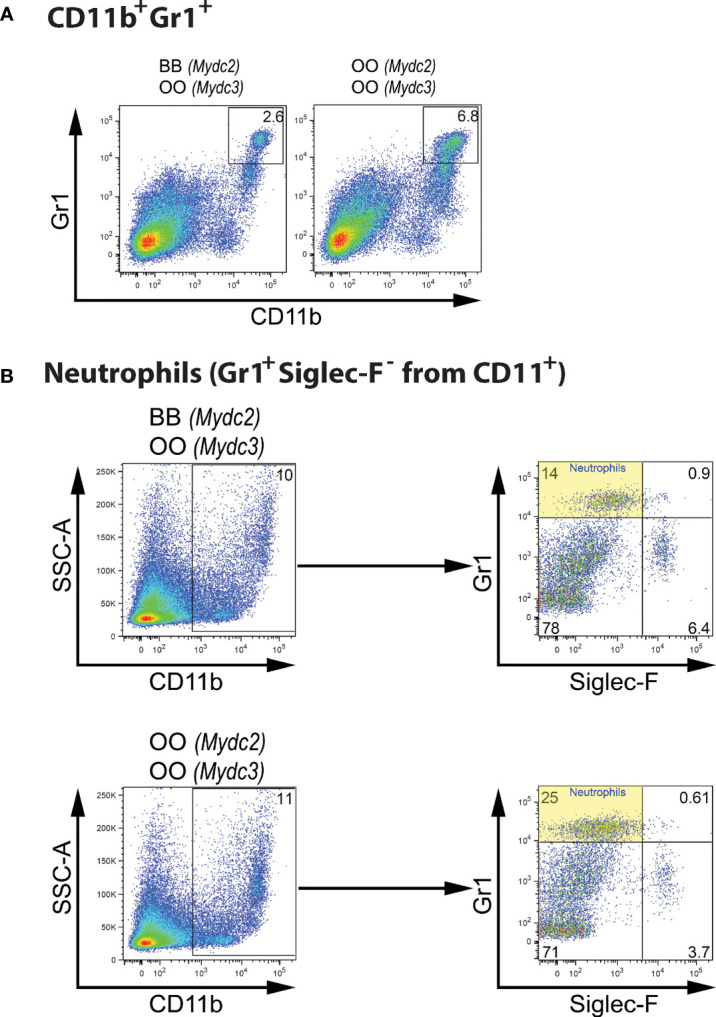
Genetic interactions influencing frequency of myeloid-derived cells. **(A)** Flow cytometry analysis of spleens of representative mice carrying combination of BB (*Mydc2*) and OO (*Mydc3*) genotypes, and OO homozygotes in both *Mydc2* and *Mydc3* showing the Gr1/CD11b cell surface marker status of individual cells. **(B)** Spleen cell gating strategy for analysis of genetic influence on neutrophil frequency. O and B indicate the presence of O20 and B10 allele, respectively.

Interaction between locus *Mydc2* linked to rs78065633 on chromosome 15 and locus *Mydc3* linked to D17Mit197 on chromosome 17 controls both frequency of CD11b^+^Gr1^+^ cells (**Table 4A** and **Figures 5A**, **6A**) and Gr1^+^Siglec-F^-^ subpopulation from CD11b^+^ cells (**Table 4B** and **Figures 5B** and **6B**) in spleen. In both interactions, higher levels of tested subpopulations were present in OO homozygotes in both *Mydc2* and *Mydc3*. The linkages were detected only in the cross B10xB10.O20, but the interactions between cross and marker were not significant (nominal *p*-value = 0.15 and 0.11, respectively).

**Table 4:** Interaction between Mydc2 and Mydc3 controls the levels of CD11b^+^Gr1^+^ cells (A) and neutrophils (Gr1^+^Siglec-F^-^ cells from CD11b^+^ cells) (B) in spleen.

A.													
Marker	Cross	Genotype	Chr.15 - rs78065633 - *Mydc2*									*p*	Bonf. corr. *p*
			BB			OB			OO				
**D17Mit197** ***Mydc3***	Both	**BB**	**2.78**	1.23	± 0.04	**2.99**	1.24	± 0.03	**3.60**	1.29	± 0.06	NS	NS
					(*n* = 16)			(*n* = 28)			(*n* = 09)		
		**OB**	**2.73**	1.22	± 0.03	**2.32**	1.18	± 0.02	**2.48**	1.20	± 0.04		
					(*n* = 31)			(*n* = 75)			(*n* = 23)		
		**OO**	**1.79**	1.12	± 0.03	**2.70**	1.22	± 0.03	**3.37**	1.28	± 0.06		
					(*n* = 27)			(*n* = 37)			(*n* = 09)		
	B10 x B10.O20	**BB**	**3.02**	1.25	± 0.09	**4.84**	1.37	± 0.05	**4.22**	1.33	± 0.06	0.001	0.008
					(*n* = 03)			(*n* = 07)			(*n* = 05)		
		**OB**	**3.62**	1.29	± 0.04	**3.23**	1.26	± 0.03	**2.28**	1.18	± 0.04		
					(*n* = 11)			(*n* = 17)			(*n* = 09)		
		**OO**	**2.26**	1.18	± 0.04	**2.38**	1.19	± 0.07	**7.86**	1.51	± 0.08		
					(*n* = 14)			(*n* = 07)			(*n* = 03)		
	B10.O20 x B10	**BB**	**2.06**	1.16	± 0.05	**2.23**	1.17	± 0.04	**2.47**	1.20	± 0.09	NS	NS
					(*n* = 13)			(*n* = 21)			(*n* = 04)		
		**OB**	**2.00**	1.15	± 0.04	**1.75**	1.12	± 0.02	**2.26**	1.18	± 0.05		
					(*n* = 20)			(*n* = 58)			(*n* = 14)		
		**OO**	**1.40**	1.07	± 0.05	**2.32**	1.18	± 0.03	**2.00**	1.15	± 0.07		
					(*n* = 13)			(*n* = 30)			(*n* = 06)		
**B.**													
**Marker**	**Cross**	**Genotype**	**Chr.15 - rs78065633 - *Mydc2***									***p***	**Bonf. corr. *p***
			**BB**			**OB**			**OO**				
**D17Mit197** ***Mydc3***	Both	**BB**	**19.37**	4.40	± 0.21	**19.50**	4.42	± 0.16	**21.55**	4.64	± 0.28	NS	NS
					(*n* = 16)			(*n* = 28)			(*n* = 09)		
		**OB**	**18.66**	4.32	± 0.15	**17.35**	4.17	± 0.10	**17.96**	4.24	± 0.18		
					(*n* = 31)			(*n* = 75)			(*n* = 23)		
		**OO**	**16.13**	4.02	± 0.16	**18.24**	4.27	± 0.14	**20.52**	4.53	± 0.28		
					(*n* = 27)			(*n* = 37)			(*n* = 09)		
	B10 x B10.O20	**BB**	**21.80**	4.67	± 0.48	**26.16**	5.11	± 0.31	**25.72**	5.07	± 0.36	0.006	0.048
					(*n* = 03)			(*n* = 07)			(*n* = 05)		
		**OB**	**23.50**	4.85	± 0.25	**20.50**	4.53	± 0.21	**17.01**	4.12	± 0.27		
					(*n* = 11)			(*n* = 17)			(*n* = 09)		
		**OO**	**17.72**	4.21	± 0.22	**19.90**	4.46	± 0.33	**35.35**	5.95	± 0.46		
					(*n* = 14)			(*n* = 07)			(*n* = 03)		
	B10.O20 x B10	**BB**	**16.29**	4.04	± 0.23	**15.68**	3.96	± 0.18	**16.80**	4.10	± 0.41	NS	NS
					(*n* = 13)			(*n* = 21)			(*n* = 04)		
		**OB**	**15.03**	3.88	± 0.18	**14.53**	3.81	± 0.11	**17.32**	4.16	± 0.22		
					(*n* = 20)			(*n* = 58)			(*n* = 14)		
		**OO**	**14.50**	3.81	± 0.22	**16.14**	4.02	± 0.15	**14.10**	3.76	± 0.33		
					(*n* = 13)			(*n* = 30)			(*n* = 06)		

Second row and column indicate the genotype of the corresponding locus. In order to obtain normal distribution required for ANOVA, the following transformations were used: CD11b^+^Gr1^+^ (% in spleen homogenates) - power of 5; % of Gr1^+^Siglec-F^-^ cells from CD11b^+^ cells – power of 2. Transformed means ± SEM are shown next to average non-transformed mean values in bold. n indicates the number of mice.

### Locus *Rsw1* on Chromosome 17 Influences Relative Spleen Weight as Well as Spleen Architecture

**Figure 7:**
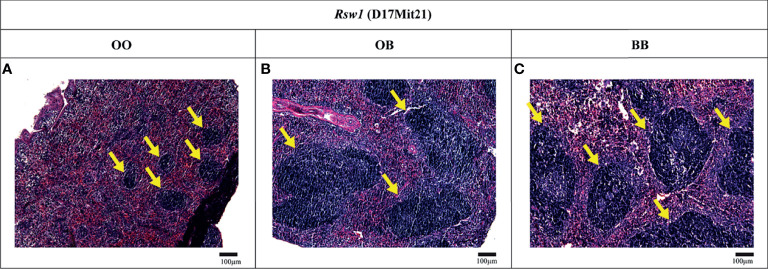
Spleen histology. Light micrographs of the hematoxylin and eosin-stained paraffin-embedded spleen sections of F_2_ hybrids between B10.O20 and B10. Indicated alleles in bold correspond to the genotype of *Rsw1* (linked with D17Mit21). White pulps regions are indicated by yellow arrows. Bars represent 100 μm. Original magnification ×10.

**Figure 8:**
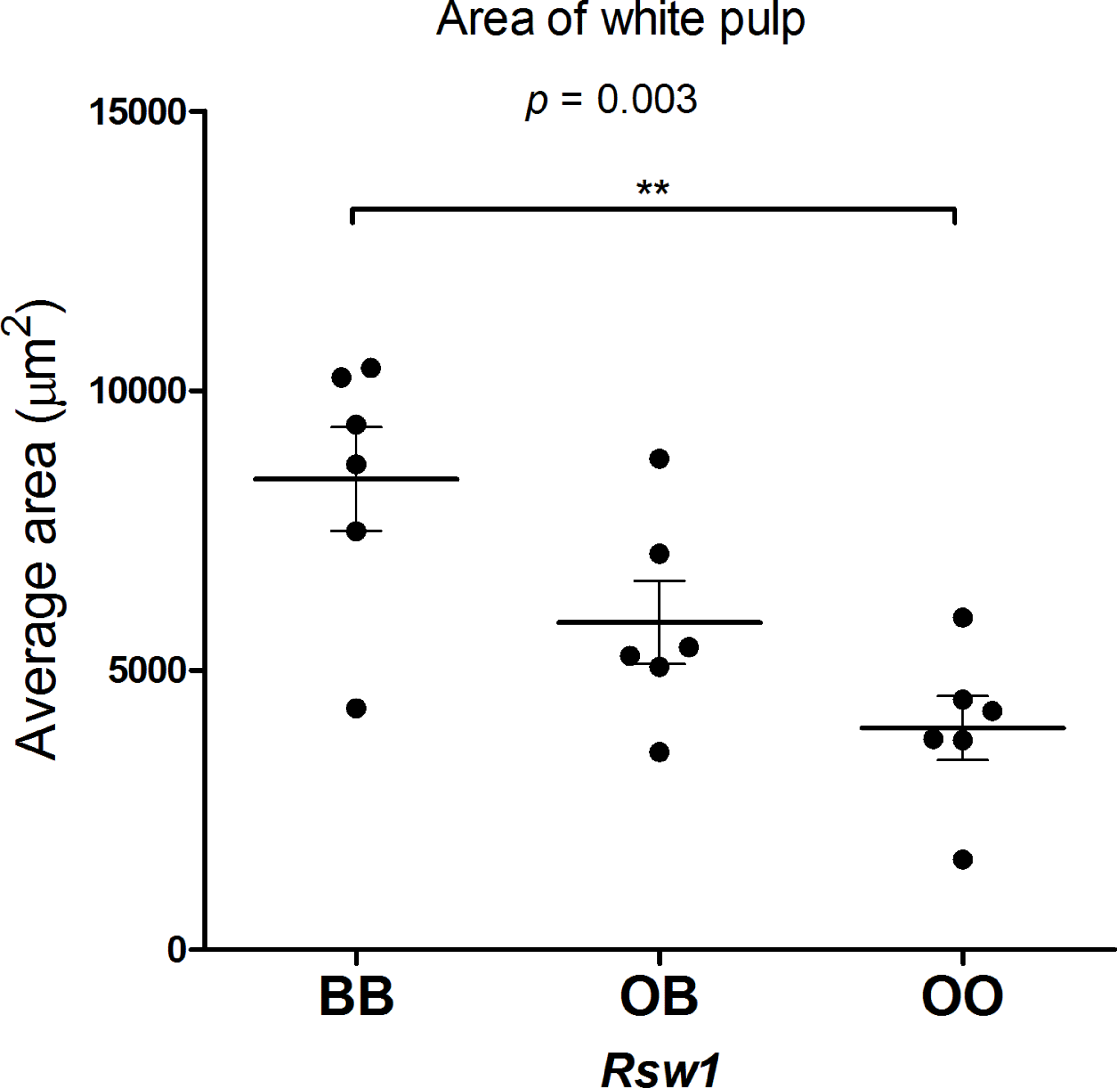
Area of white pulp in F_2_ hybrids between B10.O20 and B10. The area of the white pulp was measured using the ellipse formula a*b*π, where “a” is the major radius and “b” is the minor radius of the white pulp. Each dot represents the average area of 10 white pulps measured three times from each sample. Average age 14 weeks (median 14 weeks, min 13 weeks, max 16 weeks). Statistical analysis was performed by ANOVA, *p* = 0.0033. Bars represent the average ± SEM. **p < 0.01.

To investigate the effect of *Rsw1* locus on spleen architecture, we compared hematoxylin-eosin-stained spleen sections of F_2_ hybrids between B10 and B10.O20 with the different alleles on *Rsw1* (**Figure 7**). Interestingly, B10 homozygotes had twice larger white pulps than mice homozygous for O20 alleles (**Figure 8**). This observation correlates with the results in **Figure 1**, where frequency of lymphocytes (white pulp residents) is lower and frequency of granulocyte subsets (red pulp residents) is higher in the strain B10.O20, carrying O20 allele in *Rsw1*.

### Potential Candidate Genes

In order to identify potential candidate genes controlling the phenotypes listed in **Table 2**, we sequenced the strains B10 and O20 using NGS and identified the genetic variants between B10 and O20 in the O20-derived region of B10.O20. Then, we used a range of Bioinformatics tools to predict the effects of the detected variants on the structure and function of proteins and on RNA stability (**Table 5**). All but one (in gene *Gtpbp1*) structural differences from the C57BL/6 standard strain were of O20 origin.

**Table 5:** List of candidate genes controlling cell subpopulation frequencies in strain B10.O20.

Chr.	Position Bp	Reference genotype C57BL/6	Genotype C57BL/10	Genotype O20	Protein position of AA	Reference AA	Alteration	Type of change	Conservation score	Gene symbol		Gene name	Transcription status	Gene ID: MGI	Gene ID: NCBI
15	77955898	G/G	G/G	T/T	64	P	Q	Single AA Change	9(F)	*Foxred2*		FAD-dependent oxido-reductase domain containing 2	Known	106315	239554
15	79712097	T/T	G/T	T/T	279	V	G	Single AA Change	7(-)	*Gtpbp1*		GTP binding protein 1	Known	109443	14904
17	29139662	G/G	G/G	A/A	275	G	R	Single AA Change	9(F)	***Rab44***		RAB44, member RAS oncogene family	Known	3045302	442827
17	32925358	G/G	G/G	C/C	454	P	A	Single AA Change	7(-)	*Cyp4f13*		Cytochrome P450, family 4, subfamily f, polypeptide 13	Known	2158641	170716
17	33065434	T/T	T/T	G/G	798	D	A	Single AA Change	7(-)	*Phf8-ps*		PHD finger protein 8, pseudogene	Known	1921292	74042
	33067588	A/A	A/A	C/C	80	L	W		6(-)						
17	33960287	C/C	C/C	T/T	301	R	*	Nonsense	7(24F, 17S)	***Vps52***		Vacuolar protein sorting-associated protein 52 homolog	Known	1330304	224705
	33957014	GGT/GGT	GGT/GGT	G/G	intronic			Frameshift	ND(-)						
17	34334458	C/C	C/C	T/T	206	P	L	Single AA Change	6(-)	*H2-Eb2*		Histocompatibility 2, class II antigen E beta2	Known	95902	381091
17	34472636	T/T	T/T	A/A	293	K	M	Single AA Change	3(-)	*Btnl4*		Butyrophilin-like 4	Novel	1932036	632126
17	34508126	A/A	A/A	C/C	477	F	V	Single AA Change	6(-)	*Btnl6*		Butyrophilin-like 6	Known	1932038	624681
	34508894	T/T	T/T	C/C	337	Q	R		4(-)						
	34515534	C/C	C/C	G/G	85	E	Q		8(F)						
17	34671418	G/G	G/G	A/A	245	R	H	Single AA Change	7(-)*	***Tnxb***		Tenascin XB	Known	1932137	81877
	34692397	C/C	C/C	T/T	1681	P	L		1(-)*						
	34694339	G/G	G/G	A/A	1899	G	R		8(-)*						
	34670664	CCTT/CCTT	CCTT/CCTT	C/C	45	S	.	Deletion	9(F)						
17	35064357	G/G	G/G	A/A	235	S	F	Single AA Change	5(-)*	*Mpig6b*		Megakaryocyte and platelet inhibitory receptor G6b	Known	2146995	106722
17	36127509	G/G	G/G	T/T	64	K	N	Single AA Change	ND*	*Gm19684*		Predicted gene, 19684	Novel	5011869	100503422
17	37589717	A/A	A/A	T/T	212	V	D	Single AA Change	8(-)	*Olfr114*		Olfactory receptor 114	Known	2177497	258284
	37589987	C/C	C/C	T/T	122	R	H		7(-)				
17	42666847	G/G	G/G	A/A	535	A	V	Single AA Change	8(-)	*Adgrf4*		Adhesion G protein-coupled receptor F4	Known	1925499	78249
17	43602848	A/A	A/A	C/C	227	E	D	Single AA Change	9(F)	*Pla2g7*		Phospholipase A2, group VII	Known	1351327	27226
17	45601470	C/C	C/C	T/T	92	A	T	Single AA Change	4(-)*	*Mymx*		Myomixer, myoblast fusion factor	Known	3649059	653016
17	47467203	C/C	C/C	T/T	618	T	I	Single AA Change	2(-)*	*AI661453*		Expressed sequence AI661453	Known	2146908	224833
17	21560237	AACGCA/ AACGCA	AACGCA/ AACGCA	**ACTACA/** **ACTACA**	116	NA	TT	Multiple AA Change	1(-)	*Zfp52*		Zinc finger protein 52	Known	99199	22710
	21560742	CATT/CATT	CATT/CATT	**C/C**	285	L	.	Deletion	8(-)						
17	25125213	A/A	A/A	**AAGGC/** **AAGGC**	449			Frameshift	9(F)(5F,4S)	*Ptx4*		Pentraxin 4	Known	1915759	68509
17	32189019	CG/CG	CG/CG	**C/C**	143			Frameshift	2(-)*	*Ephx3*		Epoxide hydrolase 3	Known	1919182	71932
17	34264933	CAGCCAGCCGGAGAT/CAGCCAGCCGGAGAT	CAGCCAGCCGGAGAT/CAGCCAGCCGGAGAT	**TAAGCAGTA/TAAGCAGTA**	90	SQPEI	KQY	Multiple AA Change	4(-); 4(-); 1(-); 4(-); 6(-)		*H2-Ab1*	Histocompatibility 2, class II antigen A, beta 1	Known	103070	14961
17	35188379	TC/TC	TC/TC	T/T	4			Frameshift	ND(11F, 10S)		*Lst1*	Leukocyte specific transcript 1	Known	1096324	16988
17	35320876	GC/GC	GC/GC	G/G	40			Frameshift	ND(43F, 15S)		*H2-Q1*	Histocompatibility 2, Q region locus 1	Known	95928	15006
17	35345698	GA/GA	GA/GA	G/GA	359			Frameshift	ND(-)		*H2-Q2*	Histocompatibility 2, Q region locus 2	Known	95931	15013
17	36161580	T/T	T/T	**TAGATC/** **TAGATC**				Frameshift	ND(-)		*2410017I17Rik*	RIKEN cDNA 2410017I17	Novel	1916967	675325
17	36164977	C/C	C/C	C/CTT	365			Frameshift	ND(-)		*Gm8909*	Predicted gene 8909	Novel	3704134	667977
17	36987807	AG/AG	AG/AG	A/A	249			Frameshift	ND(23F, 7S)		*H2-M5*	Histocompatibility 2, M region locus 5	Known	95917	240095
17	37575157	TCTGTG/ TCTGTG	TCTGTG/ TCTGTG	T/T	90			Frameshift	ND(17F, 24S)		*Olfr113*	Olfactory receptor 113	Known	2177496	258286
17	38417151	TC/TC	TC/TC	T/T	80			Frameshift	ND(0F, 0S)		*Esp36*	Exocrine gland secreted peptide 36	Putative	5141873	100126765
17	38644689	GGTTT/GGTTT	GGTTT/GGTTT	G/G	75			Frameshift	ND(F)(1F, 0S)		*Esp31*	Exocrine gland secreted peptide 31	Known	5141981	100126768
	38644721	T/T	T/T	TA/TA	85			Frameshift	ND*						
17	48308000	CTA/CTA	CTA/CTA	C/C	172			Frameshift	ND(12F; 1S)		*Treml2*	Triggering receptor expressed on myeloid cells-like 2	Known	2147038	328833
18	60270068	G/G	G/G	A/A	318	R	C	Single AA Change	6(-)		*Gm4841*	Predicted gene 4841; interferon-gamma-inducible GTPase Ifgga3 protein	Known	3643814	225594
	60270559	A/A	A/A	G/G	154	I	T		8(-)						
	60270567	G/G	G/G	**C/C**	151	D	E		9(F)						
	60270654	A/A	A/A	**T/T**	122	N	K		6(-)						
	60270668	G/G	G/G	**A/A**	118	P	S		8(-)						
	60270734	C/C	C/C	**T/T**	96	E	K		5(-)						
	60270794	T/T	T/T	**C/C**	76	T	A		9(F)						
	60270814	G/G	G/G	**T/T**	69	T	N		6(-)						
	60270868	T/T	T/T	**TCCC/TCCC**	50	G	GG	Insertion	6(-)						
18	60300013	C/C	C/C	**G/G**	56	S	C	Single AA Change	5(-)		*F830016B08Rik*	RIKEN cDNA F830016B08; interferon-gamma-inducible GTPase Ifgga4 protein	Known	3588218	240328
	60300354	G/G	G/G	**A/A**	170	A	T		7(-)						
	60300671	C/C	C/C	**G/G**	275	Y	*	Nonsense	6(-)						
18	65349741	T/T	T/T	**C/C**	399	R	G	Single AA Change	8(F)		*Alpk2*	Alpha-kinase 2	Known	2449492	225638
18	57294020	A/A	A/A	**AGC/AGC**	1134			Frameshift	ND(2F)*		*Megf10*	Multiple epidermal growth factor-like domains protein 10	Known	2685177	70417

The conservation score is inferred from the ConSurf software on July 15, 2019. The conservation score ranging from 1 to 9 is followed in brackets by the type of the residue or the number of altered (F and S) residues (F functional, S structural, - neither functional nor structural, *unreliable due to insufficient data). The higher the score, the more conserved the altered residue. ND, not determined. AA, amino acid. Red in the Genotype column marks difference from the reference genotype, red in the Gene symbol column marks differential expression.

**Figure 9:**
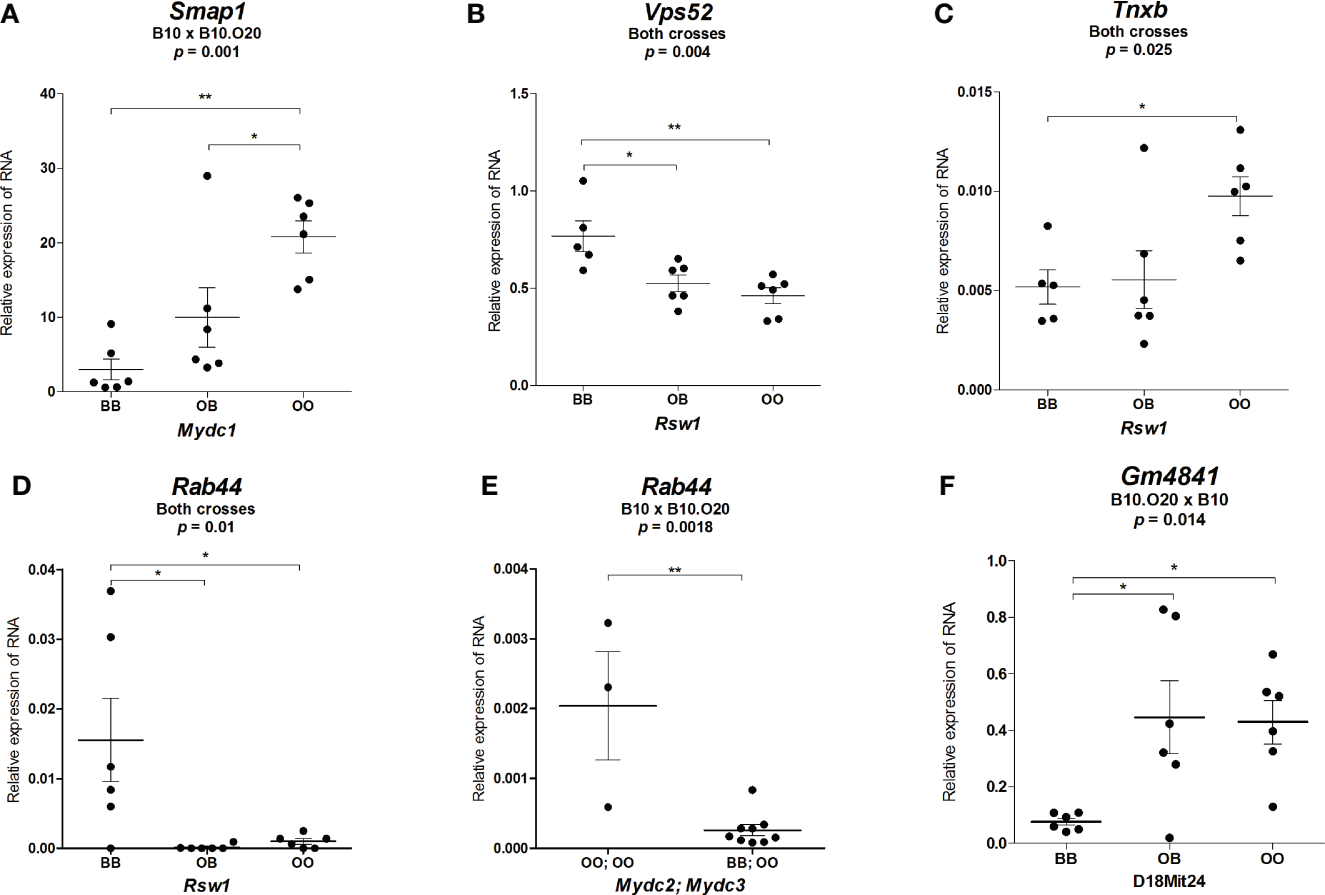
Expression of mRNA of potential candidate genes in spleens of F_2_ mice. Relative expression of a target gene versus a reference gene *Gapdh* is shown. Only genes with significant differential expression are presented. **(A)** Relative expression of *Smap1* RNA in mice carrying different alleles of *Mydc1* (chr.1). **(B)** Relative expression of *Vps52*, **(C)** *Tnxb*, and **(D)** *Rab44* RNA in mice carrying different alleles of *Rsw1*. **(E)** Relative expression of *Rab44* RNA in combined genotypes of *Mydc2* (linked with rs78065633 – chr.15) and *Mydc3* (linked with D17Mit197) corresponding to the lowest (BB; OO) and highest (OO; OO) frequency of CD11b^+^Gr1^+^ and neutrophils (Gr1^+^Siglec-F^-^ cells from CD11b^+^ cells) in the cross B10xB10.O20. **(F)** Relative expression of *Gm4841* RNA in mice carrying different alleles of D18Mit24. Statistical analysis was performed in **(A–D, F)** by ANOVA followed by Bonferroni multiple comparison test or in **(E)** by two-tailed unpaired *t*-test. p-values are as indicated. Bars represent the average ± SEM. *p < 0.05; **p < 0.01.

This analysis revealed two potential candidate genes on chromosome 15, 29 genes on chromosome 17, and four genes on chromosome 18 (**Table 5**); no polymorphisms affecting gene functions were found on chromosome 1. We chose *Foxred2* in *Mydc2* (chromosome 15); *Rab44*, *Vps52, Tnxb, Pla2g7, Ptx4, Ephx3, Lst1, H2-M5, Olfr113*, and *Treml2* in *Mydc3/Rsw1* (chromosome 17); and *Gm4841*, *F830016B08Rik*, *Alpk2* and *Megf10* on chromosome 18 for RNA expression studies. Selection of these genes for testing was based on the importance of the variation (we prioritized frameshift, nonsense mutation, and variants of highly conserved residues) in the corresponding loci. Samples of different genotypes were randomly selected based on their age. The differentially expressed genes (*Vps52*, *Tnxb*, *Rab44*, and *Gm4841*) are shown in **Figure 9**. The expression of the remaining genes was either undetectable (*Ptx4, Ephx3, H2-M5, F830016B08Rik*, and *Megf10*) or expressed without significant difference between the tested groups (*Foxred2, Lst1, Pla2g7*, *Olfr113*, *Alpk2* and *Treml2*) (**Supplementary Figure 3**).

#### *Smap1* Is a Potential Candidate Gene for *Mydc1*

Since the bioinformatics analysis of deleterious variants did not identify any candidate gene in the locus *Mydc1*, which is directly associated with the levels of CD11b^+^Gr1^+^ cells in spleen, we searched the Mouse Genome Informatics (42) for phenotypic function of the 8 genes (*4933415F23Rik*, *Mir30a*, *Mir30c-2*, *Ogfrl1*, *B3gat2*, *Smap1*, *Sdhaf4*, and *Col9a1*) located in *Mydc1* (**Supplementary Table 3**). *Smap1* (small ArfGAP [ADP-ribosylation factor GTPase activating protein]1) is the gene involved in both the hematopoietic and the immune systems (43, 44); other genes with potential influence on MDC frequencies are *Mir30a* (microRNA 30a) (45), *Mir30c-2* (microRNA 30c-2) (46), *Ogfrl1* (opioid growth factor receptor-like 1) (47), and *Col9a1* (collagen, type IX, alpha 1) (48) (**Supplementary Tables 3, 4**). These five genes were tested for differential expression. Only *Smap1* showed significant differences among mice with different genotypes. O20 (OO) homozygotes, which control higher frequency of CD11b^+^Gr1^+^, exhibited higher expression of *Smap1* RNA than both B10 (BB) homozygotes and heterozygotes (**Figure 9A**). We observed a tendency toward differential expression in *Ogfrl1* (**Supplementary Figure 3A**), but these differences were not significant, and no differential expression was found among genotypes of *Mir30a*, *Mir30c-2i*, and *Col9a1* (**Supplementary Figures 3B–D**).

#### No Potential Candidate Gene Detected in *Mydc2*

In the linkage analysis, the effect of *Mydc2* (chromosome 15) was observed only in interaction with *Mydc3* (chromosome 17). Thus, we compared expression of *Foxred2* (FAD-dependent oxido-reductase domain containing 2) in the combination of OO homozygotes in both *Mydc2* and *Mydc3* with BB homozygotes in *Mydc2* and OO homozygotes in *Mydc3*. Although there was a tendency toward differential expression, these differences were not significant (**Supplementary Figure 3E**).

#### *Vps52*, *Tnxb*, and *Rab44* Are Potential Candidate Genes for *Mydc3*/*Rsw1*

Locus on chromosome 17 is involved in control of relative spleen weight (*Rsw1*) and frequencies of CD11b^+^Gr1^+^ cells and neutrophils (*Mydc3*). *Rsw1* exhibits the main (single) gene effect, whereas the influence of *Mydc3* is observed only in interaction with *Mydc2*. Three genes—*Vps52* (Vacuolar protein sorting-associated protein 52 homolog), *Tnxb* (tenascin XB), and *Rab44* (RAB44, member RAS oncogene family)—exhibited differential expression characteristic for a single gene effect. The O20 allele of *Vps52* carries a non-sense mutation that results in a loss of 24 functional and 17 structural residues (**Table 5**). O20 homozygotes (OO) as well as heterozygotes (OB) of *Vps52* have approximately 1.6-fold lower expression than B10 (BB) homozygotes (**Figure 9B**). The O20 variant of TNXB includes a deletion of a highly conserved functional serine (S45del) with two single amino acid changes of three other residues (R245H, P1681L, and G1899R) (**Table 5**). O20 homozygotes exhibited higher *Tnxb* RNA expression than both heterozygotes and B10 homozygotes (**Figure 9C**). The O20 allele of *Rab44* carries a deleterious variant of a highly conserved functional residue (G275R); glycine in B10 is in O20 replaced by arginine (**Table 5**). The relative expression level of *Rab44* was partly similar to *Vps52*. Highest level of *Rab44* mRNA was observed in B10 (BB) homozygotes, while O20 (OO) homozygotes and heterozygotes exhibited almost no expression (**Figure 9D**). *Rab44* also exhibited differential expression in interaction between *Mydc2* and *Mydc3*. OO homozygotes in both *Mydc2* and *Mydc3* had higher expression of *Rab44* than the combination of BB homozygotes in *Mydc2* with OO homozygotes in *Mydc3* (**Figure 9E**). There were tendencies toward differential expression of genes *Lst1, Vps52* (*p* = 0.073), and *Tnxb* in interaction between *Mydc2* and *Mydc3*, but these differences were not significant (**Supplementary Figures 3F–H**).

#### *Gm4841* Is a Potential Candidate Gene for a Suggestive Linkage of Neutrophil Frequency on Chromosome 18

A suggestive linkage on chromosome 18 might influence neutrophil frequency (**Table 3**). We analyzed the RNA expression of the potential candidate genes on chr.18; only *Gm4841* (predicted gene 4841; interferon-gamma-inducible GTPase Ifgga3 protein) was differentially expressed, and B10 allele determined low RNA levels (**Figure 9F**). O20 allele of *Gm4841* differs from B10 allele in 8 single amino acid variants, all intermediate to highly conserved residues including two functional residues, with an insertion of one residue (**Table 5**).

## Discussion

### Combination of Genomes of Two Parental Strains Gives Rise to a Strain Exceeding MDC Frequencies of Both of Them

Frequencies of several spleen cell subpopulations in B10.O20 differ from both B10 and O20 (**Figure 1**). Observations of progeny whose phenotype is beyond the range of that of its parents, are frequent in multigenic traits. They were seen in many tests of immune responses of recombinant congenic strains *in vitro* (49–51) and *in vivo* (27, 52–54), and in analysis of expression QTLs of chromosome substitution strains (55). These observations reflect multiple regulatory interactions, which, in new combinations of genes, can lead to new quantitative phenotypes that exceed their range in parental strains.

### Novel Genes/Loci Controlling Differences in MDC Frequencies and in Relative Spleen Weight

Systems genetics allows identification of novel genes and mechanisms controlling complex diseases and phenotypes in a context similar to the natural population, which is also relevant to clinical traits (56). Here, we investigated the role of genetic variants in the control of frequencies of immune cell subpopulations in spleen of the strain B10.O20. This analysis revealed three loci *Mydc1*, *Mydc2*, and *Mydc3* that control frequencies of CD11b^+^Gr1^+^ and/or neutrophil cell subpopulations, the *Rsw1* locus influencing relative spleen weight, and a suggestive linkage to chromosome 18 influencing frequency of neutrophils (**Table 2** and **Figure 2**). We have also detected potential candidate genes *Smap1*, *Rab44*, *Vps52*, *Tnxb*, and *Gm4841.* All alterations changing genes’ functions have been detected in genes of O20 origin (**Table 5**). It is not surprising as the strain B10 (C57BL/10) is more genetically related to the reference strain C57BL/6. O20 is an inbred mouse strain of unknown origin. Despite several potentially deleterious mutations described here and retinal degeneration (57), O20 mice are otherwise healthy and are highly resistant to leishmaniasis (27), resistant to breast (58) and small intestinal (59) cancer, and susceptible to lung cancer (60).

We were unable to test genes *Ptx4, Ephx3, H2-M5, F830016B08Rik*, and *Megf10*, because their expression was very low or undetectable. These results are in agreement with findings of others (**Supplementary Table 5**).

#### Experimental Data and Literature Support Role of *Smap1* in Control of Frequency of CD11b^+^Gr1^+^ Cells by *Mydc1*

*Mydc1* modifies the frequency of CD11b^+^Gr1^+^ cells. Because we did not detect any genetic variants influencing gene function(s) on the chromosomal segment on chromosome 1 comprising *Mydc1*, we tested the expression of *Smap1*, which influences the hematopoietic and immune systems (43, 44) (**Table 6**). It also influences differentiation and migration of polymorphonuclear neutrophils via activation of Arf6 (77). B10 homozygotes that exhibit lower frequency of CD11b^+^Gr1^+^ cells than O20 homozygotes (**Figure 9A** and **Table 3**) also show lower level of *Smap1* expression. As we have detected neither functional polymorphism in *Smap1* gene nor extrachromosomal segment interacting with *Mydc1*, differential expression of *Smap1* is likely cis-regulated by genetic element localized outside this gene.

**Table 6:** List of differentially expressed candidate genes in the strain B10.O20.

*Gene* (Name)	Function	Connection with diseases
***Mydc1***		
*Smap1* (small ArfGAP 1)	An ARF6 GTPase-activating protein that functions in clathrin-dependent endocytosis and plays a role in blood cell proliferation and development (43). ARF6 participates in functions of polymorphonuclear leukocytes (61).	The human orthologue *SMAP1* in the 6q13 region - association with aplastic anemia (62); tumor suppressor gene: prostate cancer (63), acute myeloid leukemia (64), and colon cancer (65). Also associated with pediatric venous thromboembolism (66). *Via* ARF6 - regulation of cancer cell invasion and metastasis, as well as tumor angiogenesis and growth (67, 68).
***Mydc3***		
*Rab44* (RAB44, member RAS oncogene family	A large Rab-GTPase that contains a Rab-GTPase domain and some additional N-terminal domains (69). Rab proteins cycle between the cytosol and the membrane of its respective transport compartment and regulate budding, uncoating mobility and fusion of vesicles (70). It plays a role in osteoclast differentiation (71) and granule exocytosis in mast cells (69).	IgE-mediated anaphylaxis (69).
***Rsw1***		
*Rab44* (RAB44, member RAS oncogene family)	See ***Mydc3***	
*Vps52* (vacuolar protein sorting 52)	Part of GARP (Golgi-associated retrograde protein) and EARP (endosome-associated recycling protein) complexes. It is involved in retrograde transport of endosomes (72). *Vps52* also plays a role during embryonic development (73).	Tumor suppressor in gastric cancer (74).
*Tnxb* (tenascin-XB)	A large matricellular glycoprotein, ubiquitously expressed during late embryogenesis—probably a role in organogenesis; adult organisms—present in connective tissue in a variety of organs. It regulates bioavailability of TGFβ, participates in wound healing, has an indirect involvement in cell signaling, cell adhesion (75), influences early myeloid and lymphoid differentiation (42, 76).	Likely exerts tumor-suppressive activities (75). Polymorphism associated with multiple sclerosis, systemic lupus erythematosus, rheumatoid arthritis, ulcerative colitis, and type 1 diabetes (76).
**Chr.18**		
*Gm4841* (Predicted gene 4841)	Predicted to have GTPase activity, to be involved in cellular response to interferon-beta and defense response. Predicted to localize to endoplasmic reticulum membrane (24).	

The function of the genes and their connection with diseases is described.

#### Interaction Between *Rab44*/*Mydc3* With an Unknown Partner in Locus *Mydc2* Might Control Frequencies of CD11b^+^Gr1^+^ Cells and Neutrophils

The interaction between *Mydc2* (chromosome 15) and *Mydc3* (chromosome 17) controls frequencies of CD11b^+^Gr1^+^ cells and neutrophils (**Table 4**). Neutrophils are a subgroup of CD11b^+^Gr1^+^ cells, and the influence of genotypes on frequencies of both cell subpopulations is similar. Therefore, it is possible that the difference in frequencies of CD11b^+^Gr1^+^ cells regulated by interaction between *Mydc2* and *Mydc3* is due to the difference of its neutrophil subgroup.

In the linkage analysis, the effect of *Mydc2* (chromosome 15) was observed only in interaction with *Mydc3* (chromosome 17). Bioinformatics analysis pinpointed *Foxred2* as a potential candidate gene. However, differences in expression of *Foxred2* in mice carrying OO homozygotes in both *Mydc2* and *Mydc3*, and BB homozygotes in *Mydc2* and OO homozygotes in *Mydc3* were not significant (**Supplementary Figure 3E**). Thus, *Foxred2* is an unlikely candidate gene, although its protein activity might also be regulated by modifications or structural changes that need not alter expression.

We also tested in mice with the abovementioned combination of genotypes expression of other potential candidate genes on chromosome 17: *Lst1*, *Vps52*, *Tnxb* (**Supplementary Figures 3F–H**), and *Rab44* (**Figure 9E**), but only expression of *Rab44* exhibited epistatic control. Mouse *Rab44* mRNA is highly expressed in bone marrow. It is present in bone marrow macrophages, neutrophils, and dendritic cells (78, 79) (**Supplementary Table 6**). In spleen, Rab44^+^ cells were detected in the splenic cord in the red spleen, but were hardly detectable in the white pulp (79). In bone marrow, *Rab44* is extensively expressed in undifferentiated hematopoietic CD117^+^ (c-kit) cells and its expression decreases during differentiation of immune-related cells (79). Interestingly, *Rab44* is localized in the locus *SSC7* that controls WBC count in swine (80).

#### *Rab44*, *Vps52*, and *Tnxb* Are Potential Candidate Genes for *Rsw1* Controlling Relative Spleen Weight and Spleen Architecture

*Rsw1* (chromosome 17) controls relative spleen weight, and O20 homozygotes determined higher relative spleen weight (**Table 3**). Differences in this value were associated with differences in spleen architecture, and F_2_ mice carrying B10 homozygous *Rsw1* allele showed about twice larger white pulp than O20 homozygotes (**Figures 7**, **8**). This agrees with the differences among B10, O20, and B10.O20 mice shown in **Figure 1**, in which MDC residing in the red pulp are increased while lymphocytes residing in the white pulp are decreased in B10.O20 strain. Three genes, *Rab44*, *Vsp52* and *Tnxb* (**Table 6**), had expression characteristics compatible with a single gene effect (**Figure 9**).

*Rab44* Involvement of this gene in MDC cell development is discussed in the Interaction between *Rab44*/*Mydc3* with an unknown partner in locus *Mydc2* might control frequencies of CD11b^+^Gr1^+^ cells and neutrophils section. Our results suggest a possible role for *Rab44* in influencing the splenic architecture of mice by modifying frequencies of MDC cells.

*Vps52* is expressed in many cells of the immune system, with highest levels in mega-erythrocyte progenitor (**Supplementary Table 6**) (78). The role of *Vps52* in control of spleen weight and architecture is not clear; it interacts with *ARF6* (81) that participates in functions of polymorphonuclear leukocytes (61). This is compatible with our findings.

*Tnxb* effects include hematopoiesis, and immune and hematopoietic systems (42) (**Table 6**). Targeted mutation experiments noted an association of *Tnxb* and *Btnl4* (among other 59 genes) with enlarged spleen in uninfected mice (82, 83), which supports our findings. *Bntl4* RNA expression was not tested in our samples as the variant in this gene results in a single amino acid change of low conservation score (**Table 5**).

Exome array-based meta-analysis in a multi-ancestry samples from 25 human studies found that the rs185819 variant of *TNXB* was associated with WBC count (76). In dairy cattle, *TNXB* is associated with WBC counts and with the susceptibility to the bovine leukemia virus (84).

#### *Gm4841* Is a Potential Candidate Gene for a Suggestive Linkage of Neutrophil Frequency on Chromosome 18

Heterozygotes on chromosome 18 have lower levels of neutrophils than B10 and O20 homozygotes in the (B10.O20xB10)F_2_ hybrids. Since the Bonferroni-corrected *p*-value is 0.063, it only suggests its possible linkage to neutrophil frequency. We analyzed the RNA expression of the candidate genes on chromosome 18; only *Gm4841* exhibited differential expression (**Figure 9F**).

### Newly Detected Genes/Loci Have Their Orthologs in Human, Swine, and Cattle

*Mydc1* is localized on chromosome 1 on the segment between 22.7 and 24.7 Mbp. In the near vicinity were at the position 26.97, 26.29, and 25.75 Mbp detected loci controlling WBC, granulocytes, and monocytes, respectively, in mouse blood (8). It remains to be tested whether these loci are identical with or distinct from *Mydc1.*

Orthologous human segments of peak of linkage of *Mydc1*, *Mydc2*, and *Mydc3*/*Rsw1* are localized on 6q13, 22q12-13, and 6p21, respectively (42, 85). Interestingly, human segment 6p21 orthologous to *Mydc3*/*Rsw1* was found to control WBC (17, 19), monocyte (19), neutrophil (19), lymphocyte (17), and eosinophil (18) counts (**Table 1**); swine ortholog *SSC7* determines WBC count (23, 80). *Rab44* and *TnxB—*potential candidate genes for *Mydc3*/*Rsw1—*are involved in control of WBC count in swine (80) and cattle (84), respectively.

## Conclusion

In summary, we identified three new loci on chromosomes 1, 15, and 17 (*Mydc1, Mydc2*, and *Mydc3*) controlling the frequencies of CD11b^+^Gr1^+^ and neutrophils (Gr1^+^Siglec-F^-^ cells from CD11b^+^ cells), and we show how the interaction between the two loci *Mydc2* and *Mydc3* controls frequencies of these cells. We have also identified *Rsw1*, a novel locus controlling relative spleen weight and the histological architecture of spleen. Finally, we confirmed in *Mydc1* and *Mydc3* loci a differential expression of their potential candidate genes *Smap1* and *Rab44*, respectively. *Rsw1* contains the three potential candidate genes *Vps52*, *Rab44*, and *Tnxb.* We provide a comprehensive information about the hereditary differences in the frequencies of MDC and the size and the architecture of the spleen white and red pulps. The detected loci might play a role in cancer, autoimmune diseases, and resistance to pathogens.

CD11b^+^Gr1^+^ cells comprise several subpopulations of immune cells (86). Part of these cells, a heterogeneous group of myeloid-derived suppressor cells, plays a role in cancer, autoimmune and infectious diseases, traumatic stress, and graft-versus-host disease both in mice and in humans (87, 88). Understanding the genetic regulation of MDC might improve the personalized prevention and therapy of these diseases.

Locus *Mydc3*/*Rsw1* is orthologous to the human segment 6q21 that controls WBC count (17–19). These results could be therefore useful for human studies. It would be interesting whether human segments orthologous to *Mydc1* and *Mydc2* are also controlling frequencies of MDC.

Thus, these genes can be the focus of future studies in both mice and humans.

## Data Availability Statement

The original contributions presented in the study are publicly available. These data can be found at https://www.ncbi.nlm.nih.gov/genbank/ under the accession numbers OK040659-OK040678.

## Ethics Statement

This research complies with all relevant European Union guidelines for work with animals and was approved by the Institutional Committee of the Institute of Molecular Genetics of the Czech Academy of Sciences and by Departmental Expert Committee for the Approval of Projects of Experiments on Animals of the Academy of Sciences of the Czech Republic (permission number 93/2015).

## Author Contributions

IK, YS, VH, and ML designed the project. IK and ML wrote the manuscript. IK, YS, EJ, HH, JV, AA, and VH performed the experiments. IK, YS, EJ, VV, HS, VH, PD, and ML analyzed the data. All authors contributed to the article and approved the submitted version.

## Funding

This work was supported by GACR 16-22346S, COST Action BM1404 Mye-EUNITER, and NV19-05-00457.

## Conflict of Interest

The authors declare that the research was conducted in the absence of any commercial or financial relationships that could be construed as a potential conflict of interest.

## Publisher’s Note

All claims expressed in this article are solely those of the authors and do not necessarily represent those of their affiliated organizations, or those of the publisher, the editors and the reviewers. Any product that may be evaluated in this article, or claim that may be made by its manufacturer, is not guaranteed or endorsed by the publisher.

## Abbreviations

B10.O20, C57BL/10-*H2^pz^*—mouse strain; B10.O20xB10 cross, the B10.O20 female crossed with the B10 male; B10, C57BL/10—mouse strain; B10xB10.O20 cross, the B10 female crossed with the B10.O20 male; DARC, Duffy antigen/chemokine receptor; *Gm4841*, predicted gene—Interferon-gamma-inducible GTPase Ifgga3 protein; GWAS, Genome-wide association studies; MDC, myeloid-derived cells; *Mydc*, myeloid-derived cells—genetic locus; PBS, phosphate-buffered saline; QTL, quantitative trait loci; *Rab44*, RAB44, member RAS oncogene family; *Rsw*, relative spleen weight—genetic locus; *Smap1*, small ArfGAP 1; *Tnxb*, tenascin XB; *Vps52*, VPS52 GARP complex subunit; WBC, white blood cells.
