# Supplementary material for: Genetic Influence on Frequencies of Myeloid-Derived Cell Subpopulations in Mouse
Source: Front Immunol. 2022 Jan 26;12:760881. doi: 10.3389/fimmu.2021.760881 (PMC8826059; doi:10.3389/fimmu.2021.760881)
Supplement: Supplementary file 1 [file DataSheet_1.pdf]

Supplementary Materials

Genetic influence on frequencies of myeloid-derived cell subpopulations in mouse

Imtissal Krayem[1], Yahya Sohrabi[1], Eliška Javorková[2,3], Valeriya Volkova[1], Hynek Strnad[4], Helena Havelková[1], Jarmila Vojtíšková[1], Aigerim Aidarova[1], Vladimír Holáň[2,3], Peter Demant[5], Marie Lipoldová[1]

[1]Laboratory of Molecular and Cellular Immunology, Institute of Molecular Genetics of the Czech Academy of Sciences, Vídeňská 1083, 14220 Prague, Czech Republic; [2]Faculty of Science, Charles University, 128 44 Prague, Czech Republic; [3]Institute of Experimental Medicine of the Czech Academy of Sciences, Vídeňská 1083, 14220 Prague, Czech Republic; [4]Department of Genomics and Bioinformatics, Institute of Molecular Genetics, Academy of Sciences of the Czech Republic, Vídeňská 1083, 14220 Prague, Czech Republic; [5]Department of Molecular and Cellular Biology, Roswell Park Comprehensive Cancer Center, Buffalo, New York 14263, USA

Supplementary Table 1. List of antibodies used for immunophenotyping

Antibody	Clone	Catalog number	Company
fluorescein isocyanate (FITC)-labeled anti-CD4	GK1.5	100406	BioLegend, San Diego, CA
phycoerythrin (PE)-labeled anti-CD8	53-6.7	100708	BioLegend, San Diego, CA
allophycocyanine (APC)-labeled anti-CD3	17A2	100236	BioLegend, San Diego, CA
APC anti-mouse/human CD11b	M1/70	101212	BioLegend, San Diego, CA
PE anti-mouse F4/80	BM8	123110	BioLegend, San Diego, CA
PE anti-mouse CD14	Sa14-2	123310	BioLegend, San Diego, CA
FITC anti-mouse CD19	6D5	115506	BioLegend, San Diego, CA
FITC anti-mouse Ly-6G/Ly-6C (Gr1)	RB6-8C5	108406	BioLegend, San Diego, CA
Alexa Fluor 647 anti-mouse CD22	OX-97	126108	BioLegend, San Diego, CA
FITC anti-mouse CD40	HM40-3	09404D	BD Pharmingen
PE anti-mouse Siglec-F	E50-2440	562068	BD Pharmingen
PE anti-mouse CD86	B7-2	553692	BD Pharmingen
Pacific Blue anti-TER119	TER119	116208	BioLegend, San Diego, CA

Supplementary Table 2. List of primers used for RT PCR experiments

Gene of interest	Forward primer	Reverse primer
Smap1	TGGACACCAGAACAGATACAGTGC	CTGTGGTCTTCGAAAGTTCTCTGG
Mir30a	GCGACTGTAAACATCCTCGAC	CAGCTGCAAACATCCGACTG
Mir30c-2	TGTAAACATCCTACACTCTCAGC	AGGCAGAGAGAGTAAACAGCC
Ogfrl1	CAGTCGGCTTTGCTGTATTGCC	TGCCAGTTTCATCTCTGGCAGTC
Col9a1	TGAGACCCAGGAGAGAAACCTGTC	TCATCAGTGGTCTGGCTGGTTG
Foxred2	AGATGTTCAGTGTGCTGGCTGAC	ACTCAAAGGCTGTGGCGTTCTC
Treml2	CGGCTGGGCATTTCTACCCT	TCCATCCATGGGGGTGTTGG
Vps52	CGCTGAGCAAGATCTACCTGTC	CTCAGCAACTTCCTCGTACTGC
Lst1	TTGTGCCGGTTCAGTCAGAGAGTG	GGCATAGTCAGTGCTGAGGTCTTC
TnxB	TTGGACTCAAGGATGGGAAGCG	AGCAGCTTTGCATCAGAGACAG
Pla2g7	ATCCTCGTGTGCAGATCTGTGG	ACATGGCCCTTTCTGCCTGTTC
Rab44	GGCAGATAGAGGAAGAGCCAAGTG	AATGGGCTTCAATGCTGCTGAGG
Olfr113	TGCTCAAGTTCTCCTGCTCTGATG	AGACAGGAAATCAGCCACTCCAAC
Ephx3	CAGGGTGAGTGTTCCTCCCG	ACGCATGTCTACAGCCACGA
H2-M5	GTTTACTGGAGCTGTGGTTGCC	CAGAATTCCTGGGTTCAGCCCT
Ptx4	TCACCCAGCCAGGCAAAGAA	GGCTCTGGAAGCGAACATCC
Gm4841	TCTGTGCATCCTGCTCTGAGTG	GGTGTGTGTGTGTGTGTGTGTG
F830016B08Rik	TTACGGAGGCAGCCACTGAAAG	GGCTTCTAGCCAGATAAACTGCTG
Alpk2	TGCTTCGATGCATGATAGCTGGTC	TGGAAACAGTGCCTCCCAAGTC
Megf10	TGTGACTGCTTACCTGGCTTCAC	ATCTGCCACTGGGACACACTTC
GAPDH	AACTTTGGCATTGTGGAAGG	GTCTTCTGGGTGGCAGTGAT

Supplementary Table 3. Functions of genes localized in *Mydc1*

***Gene* (Name)**	**Function**
4933415F23Rik=Ppp1r14bl (phosphatase 1, regulatory inhibitor subunit 14B like)	Protein serine/threonine phosphatase inhibitor activity (1).
Mir30a (microRNA 30a)	Alters the expression of myeloid differentiation primary response 88 (2), controls the instability of inducible CD4+ Tregs through SOCS1 (3) and reduces the suppressive function of iTregs (3), inhibits of differentiation of Th17 (4).
Mir30c-2 (microRNA 30c-2)	Regulates proliferation, apoptosis and differentiation via the Shh signaling pathway (5), regulates macrophage-mediated inflammation (6), and is involved in Th17 differentiation (7).
Ogfrl1 (opioid growth factor receptor-like 1)	A bone-marrow cell-derived accelerator of fibrotic liver regeneration in response to G-CSF treatment. Stimulates proliferation of hepatic parenchymal cell (8).
B3gat2 (beta-1,3-glucuronyltransferase 2 = glucuronosyltransferase S)	Catalyzes the transfer of a beta-1,3 linked glucuronic acid to a terminal galactose in different glycoproteins or glycolipids (9).
Smap1 (small ArfGAP1 = small ADP-ribosylation factor GTPase-activating protein 1)	An ARF6 GTPase-activating protein that functions in clathrin-dependent endocytosis and plays role in blood cell proliferation and development (10). ARF6 participates in functions of polymorphonuclear leukocytes (11).
Sdhaf4 = *Sdh8* (succinate dehydrogenase complex assembly factor 4)	*Sdhaf4* interacts specifically with the catalytic Sdh1 subunit in the mitochondrial matrix, facilitating its association with Sdh2 and the subsequent assembly of the SDH (succinate dehydrogenase) holocomplex. It prevents neurodegeneration (12).
Col9a1 (collagen, type IX, alpha 1)	A heterotrimer of 1(IX), 2(IX), and 3(IX) polypeptide chains that fold into the triple helix c; only alpha1chain forms NC4 domain participates in electrostatic interactions with polyanionic glycosaminoglycans in cartilage (13), the loss of *Col9a1* destabilizes the trabecular bone network, and impairs myeloid cell differentiation in bone marrow (14).

Supplementary Table 4. Expression of genes in *Mydc1* in organs of uninfected mice

Gene symbol	*4933415f23rik*	*mir30a*	*mir30c-2*	*Ogfrl1*	*Smap1*	*Sdhaf4*	*Col9a1*	*B3gat2*
gene ID: MGI	1914005	2676907	3619048	1917405	2138261	1915252	88465	2389490
gene ID: NCBI	66755	387225	723964	70155	98366	68002	12839	280645
median	4.6	N/A	N/A	33.7	80.5	482	5.3	4.6
**Organs**								
Spleen	4.6 =M	N/A	N/A	40.83 >M	84.82 >M	340 <M	4.87 <M	4.64 ~M
Bone marrow	4.6 =M	N/A	N/A	203 **>3M**	366 **>3M**	323 <M	5.11 ~M	4.8 ~M
Lymph nodes	4.6 =M	N/A	N/A	67.02 >M	57.3 <M	352 <M	4.69 <M	4.64 ~M
Liver	4.6 =M	N/A	N/A	55.15 >M	81.34 ~M	749 >M	4.87 <M	4.64 ~M
**Organ with highest expression**	testis **>30M**	N/A	N/A	mammary gland **>30M**	testis **>10M**	heart **>3M**	mammary gland **>30M**	kidney **>3M**
**Expression in other organs**				dorsal root ganglia **>10M**				

Abbreviations: N/A: Not assessed. In **bold**: three fold or higher expression. Data were compiled from public database BioGPS (http://biogps.org) November 3, 2021. First column: relative units; Second column: relationship to median (M); M=median value across all samples for a single probe set. Color scale: blue: lower than median, gray: similar to median, yellow to dark orange: higher than median (the intensity of yellow color is relative to the expression level as indicated)

Supplementary Table 5: **Expression status of undetected genes (*Ptx4, Ephx3, H2-Q1, H2-M5, F830016B08Rik, Megf10)*** in spleen of healthy mice of strains DBA/2JxC57BL/6J; CD1; C57BL/6J and DBA. Data was compiled from GXD (15) and Expression Atlas (16), Dec 12, 2020.

	Ptx4	***Ephx3***	***H2-Q1***	***H2-M5***	***F830016B08Rik***	***Megf10***	***Reference***
Summary of expression	no	yes - low	yes - low	yes - low	yes - low	Very low	
DBA/2J x C57BL/6J	no	yes - low	yes - low	yes - low	yes - low	yes - low	17
CD1	no	yes - low	yes - low	yes - low	yes - low	no - below cut-off	18
C57BL6/J	no	yes - low	no	yes - low	yes - low	No	18
DBA	no	yes - low	yes - low	yes - low	yes - low	no	18

Supplementary Table 6. Expression of potential candidate genes in organs and cells of uninfected mice

Gene symbol	*Foxred2*	*Gtpbp1*	*Rab44*	*Cyp4f13*	*4921501E09Rik (Phf8-ps)*	*Vps52*	*H2-Eb2*	*Tnxb*	*Gpr115*	*Pla2g7*	*Mymx*	*AI661453*	*Zfp52*
**Chromosome**	15	15	17	17	17	17	17	17	17	17	17	17	17
gene ID: MGI	106315	109443	3045302	2158641	1921292	1330304	95902	1932137	1925499	1351327	3649059	2146908	99199
Gene ID: NCBI	239554	14904	442827	170716	74042	224705	381091	81877	78249	27226	653016	224833	22710
Median	62.7	103.0	4.6	21.8	4.6	26.7	11.8	4.6	4.6	132.9	4.9	9.0	14.4
**Organs**													
Bone marrow	68.3 >M	322.3 >3M	345.2 >30M	11.7 <M	4.6 ~M	144.3 >3M	13.6 >M	4.6 <M	4.6 ~M	483.6 >3M	4.6 <M	8.3 <M	11.1 <M
Liver	5.8 <M	79.4 <M	4.6 ~M	533.1 >10M	4.6 ~M	42.2 >M	11.7 <M	19.1 >3M	4.6 ~M	35.9 <M	4.6 <M	116.8 >10M	5.7 <M
Lymph nodes	44.4 <M	250.3 >M	4.6 ~M	30.1 >M	4.6 ~M	111.7 >3M	381.3 >30M	23.3 >3M	4.6 ~M	1179.9 >3M	4.6 <M	8.1 <M	33.3 >M
Spleen	63.9 >M	233.4 >M	5.9 >M	14.1 <M	5.4 ~M	88.3 >3M	239.0 >10M	377.4 >30M	4.6 ~M	846.9 >3M	4.6 <M	8.3 <M	71.3 >3M
**Cells**													
B-cells MZ	114.7 >M	52.3 <M	4.6 ~M	51.2 >M	4.6 ~M	176.1 >3M	530.9 >30M	4.6 ~M	4.6 ~M	108.9 <M	5.1 >M	9.0 ~M	254.6 >10M
CMP	342.2 >3M	218.3 >M	181.6 >30M	21.3 <M	4.6 ~M	161.6 >3M	15.0 >M	4.6 ~M	4.6 ~M	4.8 <M	4.6 <M	13.4 >M	63.5 >3M
DCL CD8a+	49.5 <M	173.9 >M	4.6 ~M	18.5 <M	4.6 ~M	144.7 >3M	198.6 >10M	4.6 ~M	4.6 ~M	27.2 <M	4.9 ~M	8.3 <M	20.4 >M
DCM CD8a-	211.3 >3M	374.5 >3M	4.6 ~M	58.0 >M	4.6 ~M	184.5 >3M	772.5 >30M	4.6 ~M	4.6 ~M	894.5 >3M	4.6 <M	8.9 ~M	56.4 >3M
DP B220+	404.4 >3M	159.2 >M	4.6 ~M	29.1 >M	4.6 ~M	164.4 >3M	59.3 >3M	4.6 ~M	4.6 ~M	15.5 <M	5.0 >M	9.0 ~M	27.2 >M
Gr mac1+gr1+	204.2 >3M	86.5 <M	349.8 >30M	51.6 >M	4.6 ~M	170.3 >3M	11.8 ~M	4.6 ~M	8.0 >M	1018.0 >3M	22.2 >3M	8.3 <M	21.1 >M
Stem cells HSC	269.2 >3M	146.7 >M	23.0 >3M	13.5 <M	4.6 ~M	164.4 >3M	11.8 ~M	4.6 ~M	4.6 ~M	5.3 <M	5.0 ~M	15.5 >M	65.4 >3M
Macrophage bone marrow 0hr	144.8 >M	89.9 <M	4.6 ~M	17.6 <M	4.6 ~M	143.9 >3M	12.7 >M	4.6 ~M	4.6 ~M	80.1 <M	4.6 <M	8.3 <M	11.3 <M
Mast cells	1299.2 >10M	135.2 >M	3135.7 >30M	35.6 >M	4.8 ~M	266.7 >3M	11.7 ~M	4.6 ~M	4.6 ~M	15743.0 >30M	4.6 <M	8.3 <M	25.4 >M
Mega erythrocyte progenitor	97.2 >M	110.9 >M	13.9 >3M	15.4 <M	4.6 ~M	300.5 >10M	15.4 >M	4.6 ~M	4.6 ~M	12.8 <M	7.9 >M	19.9 >M	29.6 >M
Microglia	34.9 <M	63.1 <M	4.6 ~M	31.7 >M	4.6 ~M	102.3 >3M	11.8 ~M	4.6 ~M	4.6 ~M	10278.0 >30M	4.6 <M	8.3 <M	6.0 <M
Osteoclasts	26.9 <M	81.7 <M	4.6 ~M	25.7 >M	4.6 ~M	156.5 >3M	12.0 ~M	4.6 ~M	4.6 ~M	2237.4 >10M	6.2 >M	19.7 >M	15.1 ~M
T-cells CD4+	62.1 <M	266.3 >M	4.6 ~M	33.6 >M	4.6 ~M	145.6 >3M	11.8 ~M	4.6 ~M	4.6 ~M	4.8 <M	5.0 ~M	8.9 ~M	75.8 >3M
T-cells CD8+	56.0 <M	395.4 >3M	4.6 ~M	26.6 >M	4.6 ~M	150.2 >3M	12.4 >M	4.6 ~M	6.0 >M	8.9 <M	4.6 <M	8.3 <M	78.4 >3M
T-cells foxP3+	108.6 >M	95.9 <M	4.6 ~M	15.0 <M	4.6 ~M	110.7 >3M	52.0 >3M	4.6 ~M	4.9 >M	49.5 <M	6.7 >M	10.4 >M	140.8 >3M

Gene symbol	*Ptx4*	*Ephx3*	*H2-Ab1*	*Lst1*	*H2-Q2*	*Treml2*	*Olfr114*	*H2-Q1*	*H2-M5*	*Olfr113*	*F830016B08Rik*	*Alpk2*	*Megf10*
**Chromosome**	17	17	17	17	17	17	17	17	17	17	18	18	18
gene ID: MGI	1915759	1919182	103070	1096324	95931	2147038	2177497	95928	95917	2177496	3588218	2449492	2685177
Gene ID: NCBI	68509	71932	14961	16988	15013	328833	258284	15006	240095	258286	240328	225638	70417
Median	4.6	5.9	18.1	60.6	4.6	12.4	295.0	450.0	78.1	128.4	161.0	4.6	7.1
**Organs**													
Bone marrow	4.6 ~M	5.9 >M	754.1 >30M	1368.7 >10M	8.9 >M	116.7 >3M	398.9 >M	432.6 <M	83.1 >M	197.8 >M	222.5 >M	4.6 ~M	7.0 ~M
Liver	4.6 ~M	5.9 >M	300.5 >10M	72.6 >M	13.4 >M	12.4 ~M	384.3 >M	889.9 >M	82.0 >M	132.6 >M	188.8 >M	4.6 ~M	7.0 ~M
Lymph nodes	4.6 ~M	6.6 >M	18340.6 >30M	1023.7 >10M	7.1 >M	63.6 >3M	318.0 >M	1380.9 >3M	82.0 >M	149.4 >M	168.5 >M	4.6 ~M	6.3 <M
Spleen	4.6 ~M	5.8 ~M	18036.7 >30M	1169.1 >10M	7.1 >M	104.2 >3M	866.3 >M	668.5 >M	94.4 >M	160.7 >M	239.3 >M	5.9 >M	6.6 <M
**Cells**													
B-cells MZ	4.6 ~M	5.9 ~M	1661.3 >30M	58.1 ~M	7.1 >M	26.7 >M	N/A	N/A	N/A	N/A	N/A	25.0 >3M	7.0 ~M
CMP	4.6 ~M	5.9 ~M	21.7 >M	398.9 >3M	7.1 >M	20.5 >M	N/A	N/A	N/A	N/A	N/A	4.6 ~M	7.0 ~M
DCL CD8a+	4.6 ~M	5.9 ~M	5460.4 >30M	549.2 >3M	7.1 >M	11.7 <M	N/A	N/A	N/A	N/A	N/A	4.6 ~M	13.1 >M
DCM CD8a-	4.6 ~M	5.9 ~M	16091.3 >30M	4579.7 >30M	7.1 >M	12.4 ~M	N/A	N/A	N/A	N/A	N/A	11.7 >M	7.1 ~M
DP B220+	6.4 >M	8.2 >M	3018.5 >30M	279.3 >3M	7.1 >M	13.6 >M	265.2 <M	1939.3 >3M	76.4 ~M	147.2 >M	152.8 <M	4.6 ~M	7.0 ~M
Gr mac1+gr1+	4.6 ~M	6.0 ~M	19.8 >M	4046.1 >30M	15.3 >3M	123.0 >3M	N/A	N/A	N/A	N/A	N/A	4.6 ~M	21.3 >M
Stem cells HSC	4.6 ~M	5.9 ~M	23.3 >M	880.2 >10M	7.1 >M	12.4 ~M	N/A	N/A	N/A	N/A	N/A	4.6 ~M	7.1 ~M
Macrophage bone marrow 0hr	4.6 ~M	5.2 <M	40.8 >M	3009.6 >30M	13.9 >3M	7.7 <M	N/A	N/A	N/A	N/A	N/A	4.6 ~M	5.1 <M
Mast cells	4.6 ~M	5.9 ~M	9.5 <M	2552.0 >30M	9.9 >M	12.4 ~M	324.7 >M	515.7 >M	80.9 >M	141.6 >M	165.2 >M	67.9 >10M	4.6 <M
Mega erythrocyte progenitor	4.6 ~M	5.9 ~M	13.9 <M	101.9 >M	7.1 >M	21.7 >M	N/A	N/A	N/A	N/A	N/A	4.6 ~M	8.2 >M
Microglia	4.6 ~M	5.9 ~M	9.5 <M	555.7 >3M	52.2 >10M	12.4 ~M	N/A	N/A	N/A	N/A	N/A	4.6 ~M	53.3 >3M
Osteoclasts	4.6 ~M	5.9 ~M	424.3 >10M	601.6 >3M	7.1 >M	12.4 ~M	289.9 <M	2244.9 >3M	78.7 >M	130.3 >M	162.9 >M	4.6 ~M	7.0 ~M
T-cells CD4+	4.6 ~M	5.9 ~M	11.1 <M	52.9 <M	7.1 >M	24.8 >M	313.5 >M	2150.6 >3M	79.8 >M	139.3 >M	164.0 >M	4.6 ~M	7.0 ~M
T-cells CD8+	4.6 ~M	5.9 ~M	16.2 <M	80.7 >M	7.1 >M	12.4 ~M	338.2 >M	1777.5 >3M	80.9 >M	139.3 >M	168.5 >M	4.6 ~M	7.0 ~M
T-cells foxP3+	4.6 ~M	8.3 >M	172.7 >3M	122.9 >M	8.1 >M	15.2 >M	N/A	N/A	N/A	N/A	N/A	4.6 ~M	7.0 ~M

Abbreviations: MZ: Marginal zone; CMP: common myeloid progenitor; DCL: Dendritic cells lymphoid; DCM: Dendritic cells myeloid; DP: Dendritic Plasmacytoid; Gr: Granulocytes; HSC: Hematopoietic stem cells; Mac. BM: Macrophage Bone marrow; MEP: Mega erythrocyte progenitor; N/A: Not assessed. In **bold**: three fold or higher expression. Data were compiled from public database BioGPS (http://biogps.org) July 7, 2019. First column: relative units; Second column: relationship to median (M); M=median value across all samples for a single probe set. Color scale: blue: lower than median, gray: similar to median, yellow to dark orange: higher than median (the intensity of yellow color is relative to the expression level as indicated). No data was found for*: Btnl4, Btnl6, AU023871, Gm19684, 2410017I17Rik, Gm8909, Esp31,Esp36, Gm4841*

Supplementary Figure 1. Genetic influence on frequency of myeloid cell subsets. Flow cytometric analysis of spleens of representative mice of strains B10, B10.O20 and O20 shows internal cell complexity measured by SSC-A (side scatter) and CD11b, CD40, F4/80, CD14 cell surface marker status or Gr1/CD11b cell surface marker status of individual cells.

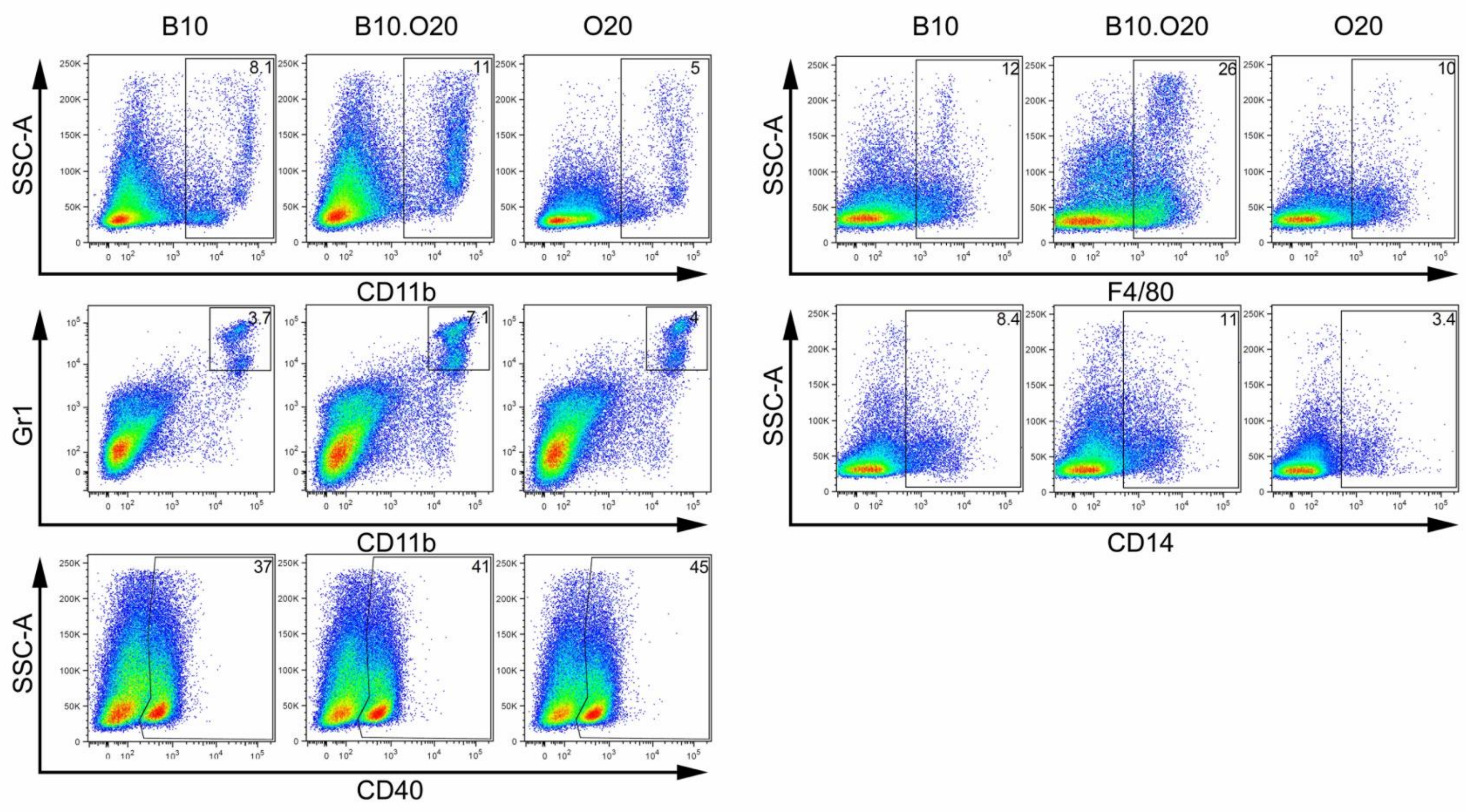

Supplementary Figure 2 Genetic influence on frequency of lymphoid cell subsets. Flow cytometric analysis of spleens of representative mice of strains B10, B10.O20 and O20 shows internal cell complexity measured by SSC-A (side scatter) and CD3, CD4, CD8, CD1 cell surface marker status, and CD4/CD3 or CD8/CD3 cell surface marker status of individual cells.

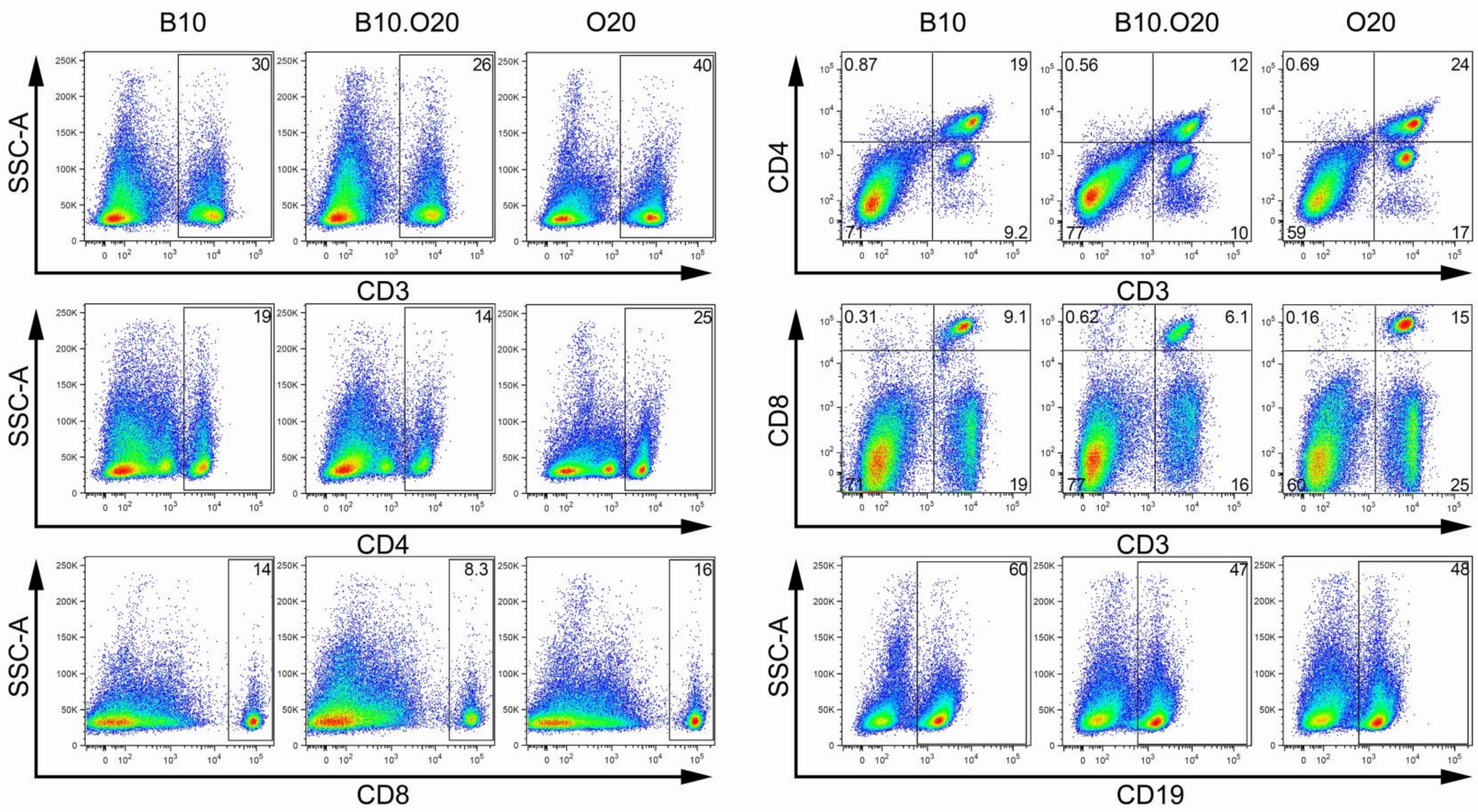

Supplementary Figure 3: **Expression of mRNA of potential candidate genes in spleen of F_2 mice.** Relative expression of a target gene versus a reference gene *Gapdh* is shown. Statistical analysis was performed by ANOVA followed by Bonferroni multiple comparison test or by two-tailed unpaired t test. *P* values are not significant (>0.05). Bars represent the average ± SEM.

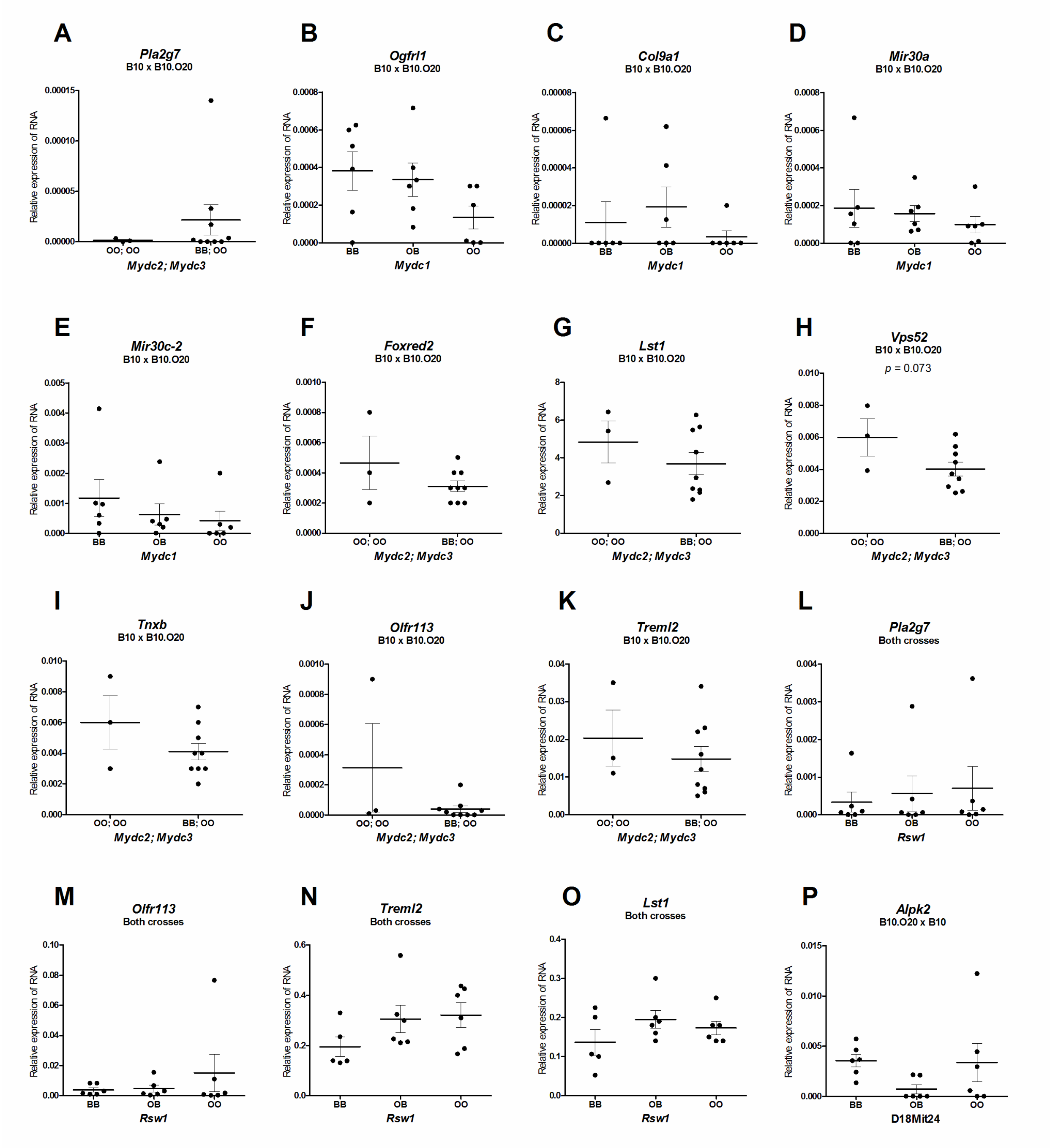

REFERENCES

1. https://www.uniprot.org/uniprot/Q14BX6 (Accessed November 1, 2021).
2. Wu Y, Sun Q , Dai L. Immune regulation of miR-30 on the Mycobacterium tuberculosis-induced TLR/MyD88 signaling pathway in THP-1 cells. *Exp Ther Med* (2017) 14(4): 3299-3303. doi: 10.3892/etm.2017.4872.
3. Zhou Y, Li Y, Lu J, Hong X, Xu L. MicroRNA30a controls the instability of inducible CD4+ Tregs through SOCS1.Mol Med Rep. (2019) 20(5):4303-4314. doi: 10.3892/mmr.2019.10666
4. Qu X, Zhou J, Wang T, Han J, Ma L, Yu H, Geng D, Fan H, Zhang Q, Hua F, et al. MiR-30a inhibits Th17 differentiation and demyelination of EAE mice by targeting the IL-21R. Brain *Behav Immun* (2016) 57: 193-199. doi: 10.1016/j.bbi.2016.03.016
5. Liu X , Li M , Peng Y, Hu X , Xu J, Zhu S, Yu Z , Han S. miR-30c regulates proliferation, apoptosis and differentiation via the Shh signaling pathway in P19 cells. *Exp Mol Med* (2016) 48(7): e248. doi: 10.1038/emm.2016.57.
6. Ceolotto G, Giannella A, Albiero M, Kuppusamy M, Radu C, Simioni P, Garlaschelli K, Baragetti A, Catapano AL, Iori E, Fadini GP, Avogaro A, Vigili de Kreutzenberg S. ; miR-30c-5p regulates macrophage-mediated inflammation and pro-atherosclerosis pathways. *Cardiovasc Res* (2017)113(13):1627-1638. doi: 10.1093/cvr/cvx157.
7. Honardoost MA, Naghavian R, Ahmadinejad F, Hosseini A, Ghaedi K. Integrative computational mRNA-miRNA interaction analyses of the autoimmune-deregulated miRNAs and well-known Th17 differentiation regulators: An attempt to discover nX8ew potential miRNAs involved in Th17 differentiation. *Gene* (2015), 572(2):153-62. doi: 10.1016/j.gene.2015.08.043
8. Yanagawa T, Sumiyoshi H, Higashi K, Nakao S, Higashiyama R, Fukumitsu H, Minakawa K, Chiba Y, Suzuki Y, Sumida K, Saito K, Kamiya A, Inagaki Y. Identification of a Novel Bone Marrow Cell-Derived Accelerator of Fibrotic Liver Regeneration Through Mobilization of Hepatic Progenitor Cells in Mice. *Stem Cells* (2019) 37(1):89-101. doi: 10.1002/stem.2916.
9. CBI https://www.ncbi.nlm.nih.gov/gene/135152
10. Kon S, Minegishi N, Tanabe K, Watanabe T, Funaki T, Wong WF, et al. *Smap1* deficiency perturbs receptor trafficking and predisposes mice to myelodysplasia. *J Clin Invest* (2013) 123(3):1123-1137. doi: 10.1172/JCI63711.
11. Gamara J, Chouinard F, Davis L, Aoudjit F, Bourgoin SG. Regulators and effectors of Arf GTPases in neutrophils. *J Immunol Res 2015* (2015). doi: 10.1155/2015/235170.
12. Van Vranken JG, Bricker DK, Dephoure N, Gygi SP, Cox JE, Thummel CS, Rutter J. SDHAF4 promotes mitochondrial succinate dehydrogenase activity and prevents neurodegeneration. *Cell Metab* (2014) 20(2):241-52. doi: 10.1016/j.cmet.2014.05.012.
13. G Vasios , I Nishimura, H Konomi, M van der Rest, Y Ninomiya, B R Olsen. Cartilage type IX collagen-proteoglycan contains a large amino-terminal globular domain encoded by multiple exons *J Biol Chem* (1988) 263(5):2324-9.
14. Probst K, Stermann J, von Bomhard I, Etich J, Pitzler L, Niehoff A, Bluhm B, Xu HC, Lang PA, Chmielewski M, Abken H, Blissenbach B, Machova A, Papadopoul,ou N, Brachvogel B. Depletion of Collagen IX Alpha1 Impairs Myeloid Cell Function. *Stem Cells* (2018) 36(11): 1752-1763. doi: 10.1002/stem.2892.

15. Beatty AE, Schwartz TS. Gene expression of the IGF hormones and IGF binding proteins across time and tissues in a model reptile. *Physiol Genomics* (2020) 52(9):423-434. doi: 10.1152/physiolgenomics.00059.2020.
16. 2. Papatheodorou I, Moreno P, Manning J, Fuentes AM, George N, Fexova S, et al. Expression Atlas update: from tissues to single cells. *Nucleic Acids Res* (2020) 48(D1):D77-D83. doi: 10.1093/nar/gkz947.
17. 3. Kelder T, Verschuren L, van Ommen B, van Gool AJ, Radonjic M. Network signatures link hepatic effects of anti-diabetic interventions with systemic disease parameters. *BMC Syst Biol* (2014) 8:108. doi: 10.1186/s12918-014-0108-0.
18. 4. Keane TM, Goodstadt L, Danecek P, White MA, Wong K, Yalcin B, et al. Mouse genomic variation and its effect on phenotypes and gene regulation. *Nature* (2011) 477(7364):289-294. doi: 10.1038/nature1041
